# Methods of Engaging Interest‐Holders in Healthcare Evidence Syntheses: A Scoping Review

**DOI:** 10.1002/cesm.70066

**Published:** 2026-01-15

**Authors:** Alex Todhunter‐Brown, Jennifer Petkovic, Christine Chang, Ursula Griebler, Ailish Hannigan, Jennifer Hilgart, Basharat Hussain, Janet Jull, Christina Koscher‐Kien, Dominic Ledinger, Barbara Nussbaumer‐Streit, Oyekola Oloyede, Eve Tomlinson, Shoba Dawson, Omar Dewidar, Sean Grant, Lyubov Lytvyn, Thomas W. Concannon, Leonila Dans, Denny John, Zoe Jordan, Evan Mayo‐Wilson, Chris McCutcheon, Francesco Nonino, Danielle Pollock, Karine Toupin April, Pauline Campbell, Joanne Khabsa, Olivia Magwood, Vivian Welch, Peter Tugwell

**Affiliations:** ^1^ School of Health & Life Sciences Glasgow Caledonian University Glasgow UK; ^2^ Bruyère Health Research Institute University of Ottawa Ottawa Ontario Canada; ^3^ MuSE Consortium member Chevy Chase Maryland USA; ^4^ Department for Evidence‐based Medicine and Evaluation University for Continuing Education Krems Krems Austria; ^5^ School of Medicine University of Limerick Limerick Ireland; ^6^ Evidence Production and Methods Directorate Cochrane UK; ^7^ School of Medicine University of Nottingham Nottingham UK; ^8^ Health Sciences University of Carleton Ottawa Ontario Canada; ^9^ School of Medicine Sefako Makgatho Health Sciences University Pretoria South Africa; ^10^ Population Health Sciences, Bristol Medical School University of Bristol Bristol UK; ^11^ Sheffield Centre for Health and Related Research University of Sheffield Sheffield UK; ^12^ Temerty Faculty of Medicine University of Toronto Toronto Ontario Canada; ^13^ College of Education University of Oregon Eugene Oregon USA; ^14^ McMaster University Hamilton Ontario Canada; ^15^ RAND Boston Massachusetts USA; ^16^ Tufts University School of Medicine Boston Massachusetts USA; ^17^ University of the Philippines Manila Philippines; ^18^ Faculty of Life and Allied Health Sciences MS Ramaiah University of Applied Sciences Bangalore India; ^19^ Joanna Briggs Institute (JBI), Faculty of Health and Medical Sciences University of Adelaide Adelaide South Australia Australia; ^20^ Department of Epidemiology UNC Gillings School of Global Public Health Chapel Hill North Carolina USA; ^21^ Ottawa Hospital Research Institute Ottawa Ontario Canada; ^22^ Unit of Epidemiology and Statistics IRCCS Istituto delle Scienze Neurologiche di Bologna Bologna Italy; ^23^ Health Evidence Synthesis, Recommendations and Impact, School of Public Health, Faculty of Health and Medical Sciences University of Adelaide Adelaide South Australia Australia; ^24^ School of Rehabilitation Sciences, Faculty of Heath Sciences University of Ottawa Ottawa Ontario Canada; ^25^ Department of Pediatrics Faculty of Medicine University of Ottawa Ottawa Ontario Canada; ^26^ Children's Hospital of Eastern Ontario Research Institute Ottawa Ontario Canada; ^27^ Institut du savoir Montfort Ottawa Ontario Canada; ^28^ American University of Beirut Beirut Lebanon; ^29^ School of Epidemiology and Public Health University of Ottawa Ontario Ottawa Canada

**Keywords:** engagement, evidence synthesis, interest‐holder, involvement, patient and public involvement, PPI, scoping review, stakeholder, systematic review

## Abstract

**Introduction:**

Engaging interest‐holders in health care evidence syntheses may make evidence syntheses more relevant, useful, and accessible. However, the best way(s) to engage interest‐holders within the evidence synthesis process remain unknown. A previous scoping review collated 291 publications that reported interest‐holder engagement in evidence syntheses, but conclusions were limited due to poor reporting. In the present scoping review, our aim was to identify and collate up‐to‐date publications focussed on interest‐holder engagement in healthcare evidence syntheses, describe reported methods of engagement, and compare the results with those from the previous review.

**Methods:**

We updated a scoping review, following JBI guidance, using a pre‐published protocol that defined all key terminology in this field. We systematically searched five electronic databases (MEDLINE, CINAHL, EMBASE, PsycInfo, and SCOPUS). Searches were conducted from January 2016 to February 2024. Records were imported into Covidence and screened by pairs of independent reviewers, including any publications that reported engagement of interest‐holders in evidence syntheses. We extracted and coded key data relating to the evidence synthesis topic and ACTIVE framework domains (who was engaged, when, and in what way). Two reviewers independently made a judgment of the comprehensiveness of the description of methods of engagement, using a “traffic‐light” system, coding evidence syntheses with comprehensive descriptions as “green,” brief or partial descriptions as “amber,” and those with few details as “red”; disagreements were resolved through discussion. Additional detailed data relating to the engagement methods were extracted from “green” evidence syntheses. Any disagreements were resolved through discussion. Data were synthesized within tables, and narrative summaries were written to provide an overview of key methods of engaging interest‐holders within the identified evidence syntheses.

**Results:**

We identified 302 publications published since the previous review. Most (272/302, 90%) reported interest‐holder engagement in a single evidence synthesis; of these, 74% (200/272) engaged patients and/or their carers, while 17% (46/272) engaged other interest‐holders only, and the remainder (26/272, 9.6%) was unclear. Over three‐quarters of the evidence syntheses were conducted either in the United Kingdom, United States, Canada, or Australia (215/272, 79%). Most often (113/272, 42%), interest‐holders were engaged at both the initial (scope and question setting) *and* final (interpretation of results) review stages (referred to as a “top and tail” approach). Nineteen percent (51/272) were judged to provide a comprehensive (“green”) description of one or more method(s) or approach(es) to engagement in an evidence synthesis, enabling detailed data extraction and description. Most: engaged patients/public members and other interest‐holder groups (30/51, 59%); used a “closed” recruitment strategy (30/51, 59%); engaged interest‐holders during the stage of interpretation of findings (39/51, 76%); had at least one interest‐holder as a co‐author (27/51, 52%). Interest‐holders generally attended meetings at which no formal methods of engagement were used. It was common to engage interest‐holders in multiple activities throughout the review process.

**Discussion/Conclusion:**

Our international team from the MuSE consortium has updated a previous scoping review, compiling the latest evidence on interest‐holder engagement in evidence syntheses. We collated 302 publications and described the methods of interest‐holder engagement reported in 51 evidence syntheses that we judged provided the most comprehensive information. Interest‐holders have been involved at all stages of the process, using a wide range of engagement approaches, but with no clear patterns linked to the type or focus of evidence syntheses. Most commonly, patients/public and professional interest‐holders were both engaged, but around one‐quarter of our examples only engaged patients/public members, and a small number only engaged professional interest‐holders. We identified some distinct engagement strategies and have used these to inform a potential decision tool to support the selection of engagement strategies. We propose recommendations in relation to the conduct and reporting of interest‐holder engagement in evidence syntheses and future research to advance this field.

## Summary

In health care, evidence syntheses bring together the results of research studies to inform clinical practice and policy. Engagement of interest‐holders (including patients, public, carers, health professionals, policy makers, and others) in evidence syntheses is thought to make evidence syntheses more useful and usable, but the best ways of engaging interest‐holders are not known. We used recognized methods for conducting high‐quality research to bring together evidence syntheses that report interest‐holders' engagement. We described all aspects of engagement. We found 302 relevant papers that had been published since 2016 (a previous study brought together papers published before 2016). Most papers were from the United Kingdom, the United States, Canada, or Australia. Ninety percent of the papers reported interest‐holder engagement in a single evidence synthesis. In three‐quarters of these patients and carers were engaged. Fifty‐one papers gave a comprehensive description of interest‐holder engagement, and we explored these in more detail. This helped us to identify various ways of engaging interest‐holders. We brought these different ways together and made a diagram to help people make decisions about how they could plan interest‐holder engagement in a future evidence synthesis. Using our findings, we also made some recommendations to help improve future interest‐holder engagement in evidence syntheses.

## 1 Background

Evidence syntheses are central to evidence‐informed practice, providing reliable and credible information to support healthcare decisions, policies, and future research [1, 2]. Evidence syntheses identify and compile data that address specific research questions using a variety of rigorous methodological approaches [3]. There is increasing recognition towards engaging a range of interest‐holders (as defined in Box 1) in healthcare evidence syntheses as they can enhance the relevance and applicability of the findings while addressing barriers to evidence implementation [10, 11]. However, uncertainty remains regarding optimal methods to ensure meaningful engagement to produce evidence syntheses that are most useful, relevant, and accessible [12].

Box 1 **Summary of key terms used in this paper (and fully defined elsewhere [4, 5]).**

**Evidence syntheses:** Research studies that synthesize the research evidence to address health care‐related questions. They use rigorous, explicit, and transparent methods. There are a range of different types of evidence syntheses; definitions of quantitative systematic reviews, qualitative evidence syntheses, rapid reviews, realist reviews, scoping reviews, mixed method reviews, and living reviews are provided elsewhere [4].

**Interest‐holders:** “groups with legitimate interests in the health issue under consideration. The interests arise and draw their legitimacy from the fact that people from these groups are responsible for or affected by health‐related decisions that can be informed by research evidence.” This term has been introduced to replace the word “stakeholder” which may be perceived as disrespectful due to colonial connotations, and to replace other alternatives (e.g., knowledge users, consumers, partners, etc.) as all of these are considered to have limitations; full justification and explanation is provided elsewhere [6].

Eleven broad groups of interest‐holders have been identified (referred to as the “11 P's” [7]). These include patients and caregivers, the public, providers of care, policy makers, program managers, payers of health research, payers of health services, peer review editors, product makers, producers and commissioners, and principal investigators. These have been fully defined elsewhere [4]. Within this paper, the nine categories of providers of care, policy makers, program managers, payers of health research, payers of health services, peer review editors, product makers, producers and commissioners, and principal investigators are sometimes collectively referred to as “professionals” to denote their employed roles, while the two categories of patients and caregivers and public are collectively referred to as “patients/public.”

**Engagement:** A bi‐directional relationship, or collaboration, between interested people and groups and a research team that results in informed decision‐making about the selection, conduct, and use of research [8, 9]. We use this term in preference to alternatives which are in common usage in some parts of the world (e.g., involvement). It is important to note that the term involvement (often “patient and public involvement” or PPI) is used in other parts of the world (e.g., UK) with the same meaning. Engagement in research refers to research being carried out “with” or “by” interested groups (rather than research carried out “about” or “for” interested people or groups). Co‐production and other terms starting with co‐ (e.g., co‐creation) are considered to be related to, but to potentially go beyond, engagement. Definitions of key terms related to engagement, and notes relating to our preferred terms, are presented elsewhere [4].

**Interest‐holder recruitment:** Two broad approaches to the identification and recruitment of interest‐holders to be engaged in an evidence synthesis have been defined. We use these terms:

**Open recruitment:** provision of “opportunities for involvement through advertisement to the general population, allowing anyone to volunteer to get involved. Open recruitment may result in ‘fixed’ membership, where, once group members have volunteered, the membership remains the same, or in ‘flexible’ membership, where different people attend different events or contribute to different activities” [5].

**Closed recruitment:** strategies which “focus on inviting only specific people to participate. Closed strategies include invitation of known individuals or recognized experts, recruitment from membership of an existing group, or purposive sampling to achieve representation of people with key pre‐determined characteristics, experience or expertise” [5].

To address this uncertainty, the MuSE Consortium is conducting a series of evidence syntheses to collate the most up‐to‐date evidence relating to engagement of interest‐holders. An overview of this work has been presented elsewhere [4, 7]. Definitions of key terms used within this series of papers relating to interest‐holder engagement in evidence syntheses have been published in full elsewhere [4, 6] and are briefly summarized in Box 1.

A previous scoping review has collated 291 evidence syntheses, published up until 2016, which described the engagement of interest‐holders and explored the range of methods of engagement [12]. Given key developments in this field since this previous scoping review, including the establishment of best practice standards and increasing mandatory requirements from research funders for patient and/or public engagement [13, 14], one of the MuSE evidence syntheses was this planned update of the previous scoping review [7, 15].

## 2 Objectives

As stated in our protocol [15], our goal was to update a previous scoping review that collated and summarized information published up to 2016 relating to the different ways in which interest‐holders have been engaged in evidence syntheses [12].

The objectives of this updated scoping review were to:

1. Identify and collate resources that describe the engagement of interest‐holders in evidence syntheses;
2. Describe reported methods to engage interest‐holders in evidence syntheses, including:
  a. Who was engaged (type and characteristics of interest‐holder)?
  b. How were they invited to be engaged?
  c. Where were they engaged (geographical and cultural context)?
  d. Why were they engaged (what was the aim of engagement)?
  e. What did they do (how they were engaged)?
  f. When in the review process were they engaged?
  g. What level of influence, or control, did they have in decision‐making?
  h. Were there processes, strategies, or tools to support engagement and evaluation of impact (e.g., training, ethical approval, compensation, reporting frameworks, evaluation strategies)?

In addition, we sought to compare the results from the previous review with those arising from the newly identified evidence syntheses within the updated review.

## 3 Methods

We used a similar methodological approach to the previous review [12, 16], conducting a broad scoping review, followed by a descriptive synthesis of publications that provide the most comprehensive description of methods of engaging interest‐holders. We followed JBI guidance [17, 18] for scoping review methods and reported according to guidance for reporting of scoping reviews (PRISMA‐ScR [19]). Our full protocol was pre‐published and describes all details of our methods [15]. Deviations from protocol are reported in Appendix 1. A description of the engagement of interest‐holders in this review is reported using the GRIPP2 tool [20] in Appendix 2, with review author identification as interest‐holders provided in Appendix 3.

### 3.1 Searching and Selection of Studies

#### 3.1.1 Search Strategy

We conducted a comprehensive database search (MEDLINE (OVID), CINAHL (EBSCO), EMBASE (OVID), PsycInfo (OVID), and SCOPUS). Search strategies are listed in Appendix 4. Search dates were from January 2016 to February 2024.

#### 3.1.2 Eligibility Criteria

Publications eligible for inclusion were any documents, in any language, that reported engagement of interest‐holders in evidence syntheses (for detailed definitions see protocol [15]). This included:

- Evidence syntheses relating to any health and/or social care topic that reported engagement of interest‐holders, including those conducted to inform guidelines (where these report explicit methods). Evidence syntheses could address any type of healthcare‐related question (e.g., intervention effectiveness, prevalence, diagnostic test accuracy, patient experiences, volume and nature of evidence, evidence gaps), within any context (i.e., any geographical location or specific setting).
- Methods studies, commentaries, or supplementary material or accompanying articles to evidence syntheses in which the engagement of interest‐holders is described.

We excluded publications from 2015 or earlier, abstracts with insufficient information, and protocols for systematic reviews.

As we had multiple reviewers applying eligibility criteria, decision support trees were used to support consistent application of eligibility criteria and reasons for exclusion (see Appendix 5).

#### 3.1.3 Study Selection

Search results were screened in Covidence, following removal of duplicates. Two independent reviewers (from a team of 20 reviewers) applied the selection criteria to titles/abstracts, and then to full papers. Disagreements were assessed by a third reviewer, with discussion between reviewers if necessary to reach a decision.

### 3.2 Data Extraction and Coding

Two independent reviewers (from a team of 17 reviewers) extracted data on all included publications using the data extraction tool within Covidence, with disagreements resolved through discussion. Data extraction and coding included:

- Bibliographic information.
- Type of publication (i.e., methods paper, systematic review, other)
- Stated aim/objective
- For systematic reviews:
  ◦ Topic/focus of systematic review
  ◦ Methodological focus/study methodology
  ◦ Type of evidence synthesized
  ◦ Why were they engaged (aim of engagement)
  ◦ Who was engaged? (categorized as patients or public members (including carers or family members) or other interest‐holders (professionals); number of interest‐holders engaged)
  ◦ Where were they engaged?
  ◦ When were interest‐holders engaged? (Phase of review)
  ◦ How were interest‐holders engaged

Full details of data extraction domains and coding are available in Appendix 6. These domains and codes were built on the previous scoping review [12], the ACTIVE Framework [5], and the lived experiences of interest‐holders from the MuSE Consortium who contributed to the protocol development.

In addition, the independent reviewers made a judgment of the comprehensiveness of the description of methods of engagement, using a “traffic light” categorization system developed and used for the previous review [12, 16]. Publications judged to provide a comprehensive description of one (or more) method or approach to interest‐holder engagement in a single evidence synthesis were coded as “Green”; evidence syntheses judged to provide a brief or partial description were coded as “Amber” and those providing few details were coded as “Red.” Full definitions of the traffic light categorizations and examples from the previous scoping review are provided in Table 1. Where there were disagreements between “Amber” and “Red” categorizations, the decision was made by the first author (A. T.‐B.). Where one reviewer had categorized an evidence synthesis as “Green” and another had selected “Amber” or “Red,” consensus was reached through discussion between members of the review team.

**Table 1:** Traffic‐light categorization of comprehensiveness of description of interest‐holder engagement.

Traffic‐light category	Definition (from Pollock et al. [12])	Example paper, selected from Cochrane reviews included in the previous version of the scoping review, and justification for judgment.
**Green**	Comprehensive description of one (or more) method or approach to engagement. Includes information relating to who was engaged, when they were engaged and what they did. Description sufficient to enable replication, although some details may still be missing (e.g., interest‐holders may be presented as “patients” or “professionals” but further details according to the type of interest‐holder are not provided; detailed meeting agendas are not essential).	[21, 22] The published Cochrane review refers to “patient partners,” but the accompanying paper provides a comprehensive description of who was engaged and in what way.
**Amber**	Brief or partial description of one (or more) method or approach to engagement. Description sufficient to enable partial replication. Provides more information than the limited details for a “red” classification, but lacks sufficient details to be classified as “green.” For example, one may describe who was engaged and state the role or activity (e.g., “reviewing” or “discussing”) without providing further details.	[23] States “Members of the public (parent carers of children with neurodisability) contributed to this review by suggesting the topic, refining the research objectives, interpreting the findings, and reviewing the plain language summary.” Details of the parent carers are provided, but there is no further information relating to engagement.
**Red**	Few details provided and/or inadequate description of the method or approach. Description is generally limited to a few words and not more than two sentences, such as a statement saying that there was a patient advisory group, but with very few details about who was engaged and what they did. Description is insufficient to enable replication.	[24] States (under “Declaration of interests”: “One review author (CA) has endometriosis and is a member of Endometriosis UK.” No further details relating to the engagement of this (patient) review author were provided.

Studies judged to be in the “green” category for the comprehensiveness of the description of methods of engagement had additional data extraction and coding, including details relating to:

- How were interest‐holders invited to be engaged?
- Who was involved (Type of interest‐holders, based on the 11 “P's” [7]; PROGRESS‐Plus characteristics [25]; geographical location; country income level)
- What happened when? (Methods, timing, amount of engagement at different review stages; see protocol for categories and definitions)
- Level of engagement (see protocol for categories and definitions)
- Ethical approval/statement
- Acknowledgment (e.g., authorship) and compensation (e.g., payment of expenses, financial compensation, other incentives)
- Tool or method of reporting engagement
- Processes to facilitate engagement
- Reflexivity
- Disclosure of conflicts of interest

These data were extracted by 1 reviewer and checked by a second reviewer (with 12 reviewers working in pairs). Appendix 6 provides further details of data extraction domains and codes.

### 3.3 Quality Assessment of Included Studies

In line with guidance for scoping reviews [26], we did not conduct a formal assessment of the methodological quality or risk of bias of the studies included in this review.

## 4 Data Synthesis

We synthesized data for the whole group of included studies within tables and charts, with brief descriptive statements. For the group of evidence syntheses judged as “green” we summarized key characteristics of individual evidence syntheses and used the ACTIVE framework [5] to describe key features relating to engagement. We grouped “green” evidence syntheses according to the type of evidence synthesis and produced narrative summaries of what happened at which stage in the review process, providing an overview of the key ways of engaging interest‐holders within different types of evidence synthesis. We held team meetings to reflect on patterns or approaches to engagement, and how reviewers may have made informed choices about the methods of engagement to use within a planned review. We created tables comparing the results of this updated scoping review with the results of the previous scoping review.

## 5 Results of the Search

**Figure 1:**
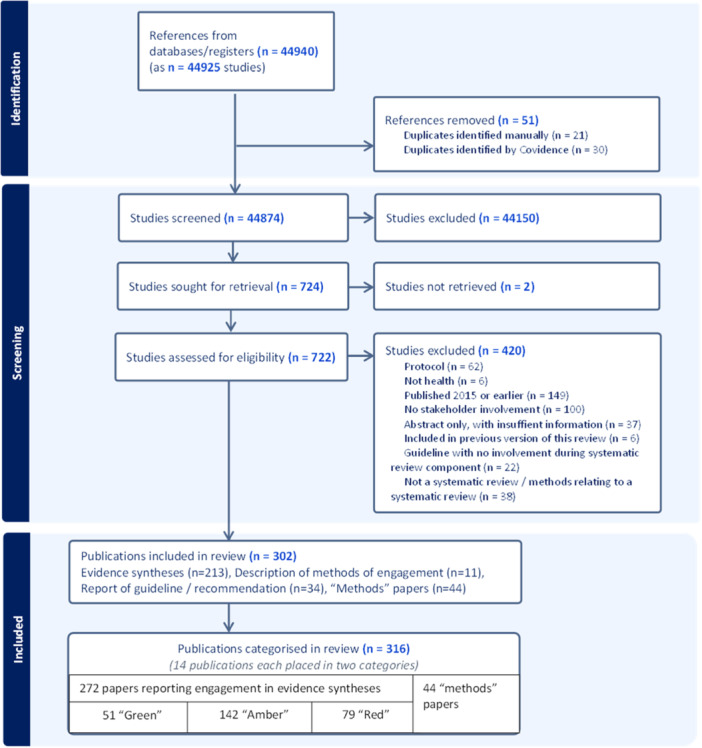
Results of the search.

Figure 1 summarizes the results of the search. We identified 302 publications (with 311 references) [5, 12, 27, 28, 29, 30, 31, 32, 33, 34, 35, 36, 37, 38, 39, 40, 41, 42, 43, 44, 45, 46, 47, 48, 49, 50, 51, 52, 53, 54, 55, 56, 57, 58, 59, 60, 61, 62, 63, 64, 65, 66, 67, 68, 69, 70, 71, 72, 73, 74, 75, 76, 77, 78, 79, 80, 81, 82, 83, 84, 85, 86, 87, 88, 89, 90, 91, 92, 93, 94, 95, 96, 97, 98, 99, 100, 101, 102, 103, 104, 105, 106, 107, 108, 109, 110, 111, 112, 113, 114, 115, 116, 117, 118, 119, 120, 121, 122, 123, 124, 125, 126, 127, 128, 129, 130, 131, 132, 133, 134, 135, 136, 137, 138, 139, 140, 141, 142, 143, 144, 145, 146, 147, 148, 149, 150, 151, 152, 153, 154, 155, 156, 157, 158, 159, 160, 161, 162, 163, 164, 165, 166, 167, 168, 169, 170, 171, 172, 173, 174, 175, 176, 177, 178, 179, 180, 181, 182, 183, 184, 185, 186, 187, 188, 189, 190, 191, 192, 193, 194, 195, 196, 197, 198, 199, 200, 201, 202, 203, 204, 205, 206, 207, 208, 209, 210, 211, 212, 213, 214, 215, 216, 217, 218, 219, 220, 221, 222, 223, 224, 225, 226, 227, 228, 229, 230, 231, 232, 233, 234, 235, 236, 237, 238, 239, 240, 241, 242, 243, 244, 245, 246, 247, 248, 249, 250, 251, 252, 253, 254, 255, 256, 257, 258, 259, 260, 261, 262, 263, 264, 265, 266, 267, 268, 269, 270, 271, 272, 273, 274, 275, 276, 277, 278, 279, 280, 281, 282, 283, 284, 285, 286, 287, 288, 289, 290, 291, 292, 293, 294, 295, 296, 297, 298, 299, 300, 301, 302, 303, 304, 305, 306, 307, 308, 309, 310, 311, 312, 313, 314, 315, 316, 317, 318, 319, 320, 321, 322, 323, 324, 325, 326, 327, 328, 329, 330, 331, 332, 333, 334, 335] that met our criteria for inclusion.

## 6 Characteristics of Included Publications

Characteristics of the included studies are provided in the Table of included studies, Supporting Information S1, and a brief summary is described below.

Two hundred and seventy‐two publications (272/302, 90.1%) reported engagement of interest‐holders in a single evidence synthesis. The remaining publications (30/302, 9.9%) did not describe engagement in a single evidence synthesis but were judged to have some relevance to interest‐holder engagement in evidence syntheses (referred to as “methods” papers). Fourteen of the 272 evidence syntheses reporting engagement in a single evidence synthesis were also considered to provide information that met our criteria for “methods” papers. Consequently, within this scoping review, we include 302 unique publications, of which 272 report engagement in a single evidence synthesis and 44 of which are “methods” papers (see Table 2).

**Table 2:** Types of included papers.

Type of publication	Sub‐category	Updated review	
Evidence syntheses relating to any health and/or social care topic that reported a case of engagement of interest‐holders in a single evidence synthesis, within a publication, as per sub‐categories.	Evidence Syntheses	226	74.8%
	Paper reporting a guideline or recommendation	34	11.2%
	Papers specifically describing details of a case of interest‐holder engagement in an evidence synthesis	12	4.0%
	*Reports of engagement in a single evidence synthesis sub‐total*	*272*	*90.1%*
Publications providing an account or description of engagement, or methods of engagement, relevant to evidence syntheses (“Methods” papers)	“Methods” papers (unique)	30	9.9%
	Publications judged to report a case of engagement *and* to be a “methods” papers	14	(4.6%)*
	*“Methods” papers sub‐total*	44	(14.6%)*
	***Total unique publications***	**302**	**100%**
	*Total publications categorized in review*	316	(104.6%)*

* Includes 14 publications which are also counted within the above Evidence Synthesis categories, taking the total publications categorized to over 100%.

### 6.1 Overview of All Publications Reporting Engagement in a Single Evidence Synthesis (*n* = 272)

#### 6.1.1 Interest‐Holders Engaged

Almost three‐quarters (200/272, 73.5%) of the evidence syntheses involved patients or public members (including carers or family members) within the evidence synthesis process (with or without other professional, interest‐holders), while 16.9% (46/272) involved other interest‐holders only (e.g., health professionals, academic experts, representatives of patient organizations). In around one‐tenth of the included evidence syntheses (26/272, 9.6%), it was not clear who the interest‐holders engaged in the review were, and whether this included patients or the public (see Table 3).

**Table 3:** Interest‐holders engaged.

Interest‐holders engaged	Reports of interest‐holder engagement in a single evidence synthesis	
Patients/carers/family/public members (with or without other interest‐holders)	200	73.5%
Other interest‐holders (professionals) only	46	16.9%
Unclear	26	9.6%
Total	272	100.0%

#### 6.1.2 Country

Most evidence syntheses engaged interest‐holders from English‐speaking countries, with 40.1% (109/272) from the United Kingdom, 19.5% (53/272) from the United States, 13.2% (36/272) from Canada, and 6.2% (17/272) from Australia. Seventeen (17/272, 6.2%) evidence syntheses each engaged interest‐holders from multiple countries, of which three included interest‐holders from African countries (see Table 4).

**Table 4:** Countries from which interest‐holders engaged.

Country	Reports of interest‐holder engagement in a single evidence synthesis	
UK	109	40.07
USA	53	19.49
Canada	36	13.24
Australia	17	6.25%
Ireland	15	5.51%
Netherlands	3	1.10%
Germany	3	1.10%
Italy	4	1.47%
France	0	0.00%
Spain	2	0.74%
Japan	1	0.37%
Switzerland	1	0.37%
Austria	0	0.00%
China	1	0.37%
Denmark	1	0.37%
Saudi Arabia	0	0.00%
Belgium	0	0.00%
Brazil	1	0.37%
New Zealand	1	0.37%
Norway	0	0.00%
Portugal	2	0.74%
Sweden	2	0.74%
Multiple countries	17	6.25%
One paper from each of Argentina, Malawi, and Singapore	3	1.10%
Total	272	100%

#### 6.1.3 Focus of the Review

Almost two‐thirds (169/272, 62.1%) of the included evidence syntheses were judged to be focussed on one of the International Statistical Classification of Diseases and Related Health Problems 11th Revision (ICD‐11) categories. Most frequently, this was “mental, behavioural or neurodevelopmental disorders” (60/272, 22.1%) and neoplasms (38/272, 14.0%). Evidence syntheses that did not fit one of the ICD‐11 categories included those focussed on a specific intervention (45/272, 16.5%) or research methods (32/272, 11.8%). Almost one‐tenth (26/272, 9.6%) were unable to be categorized within any of these groups and focussed on, for example, areas such as service delivery, teaching, data protection, and criminal justice (see Table 5).

**Table 5:** Focus of the included evidence syntheses.

Focus of evidence synthesis	Reports of interest‐holder engagement in a single evidence synthesis	
01 Certain infectious and parasitic diseases	4	1.5%
02 Neoplasms	38	14.0%
03 Diseases of the blood and blood‐forming organs	3	1.1%
04 Diseases of the immune system	1	0.4%
05 Endocrine, nutritional, and metabolic diseases	6	2.2%
06 Mental, behavioral, or neurodevelopmental disorders	60	22.1
07 Sleep‐wake disorders	0	0.0%
08 Diseases of the nervous system	15	5.5%
09 Diseases of the visual system	2	0.7%
10 Diseases of the ear or mastoid process	0	0.0%
11 Diseases of the circulatory system	1	0.4%
12 Diseases of the respiratory system	7	2.6%
13 Diseases of the digestive system	3	1.1%
14 Diseases of the skin	0	0.0%
15 Diseases of the musculoskeletal system or connective tissue	10	3.7%
16 Diseases of the genitourinary system	2	0.7%
17 Conditions related to sexual health	1	0.4%
18 Pregnancy, childbirth, or the puerperium	4	1.5%
19 Certain conditions originating in the perinatal period	1	0.4%
20 Developmental anomalies	0	0.0%
21 Symptoms, signs, or clinical findings, not elsewhere classified	0	0.0%
22 Injury, poisoning, or certain other consequences of external causes	0	0.0%
23 External causes of morbidity or mortality	1	0.4%
24 Factors influencing health status or contact with health services	10	3.7%
Interventions	45	16.5%
Other	26	9.6%
Research methods	32	11.8%
Total	272	100%

*Note:* 01–24 are ICD‐11 categories [334].

#### 6.1.4 Stage of the Review Process

Most commonly (113/272, 41.5%), interest‐holders were involved in a “top and tail” approach, with engagement both at the start of the review process, during the planning stage, and at the end, interpreting the results after the evidence had been synthesized, but the stage of the review process at which interest‐holders were involved was unclear in around one quarter of evidence syntheses (67/272, 24.6%) (see Table 6).

**Table 6:** Stage of the evidence synthesis process at which interest‐holders were engaged.

Stage of evidence synthesis process	Reports of interest‐holder engagement in a single evidence synthesis	
1. Setting scope/review questions	12	4.4%
2. Interpreting results after review completion	39	14.3%
1 and 2 (“Top and tail” approach)	113	41.5%
3. Throughout/within the review process	36	13.2%
Unclear	67	24.6%
Other (searching)	5	1.8%
Total	272	100%

#### 6.1.5 Comprehensiveness of Description of Method or Approach to Engagement

Table 7 shows the assigned judgments of the comprehensiveness of the description of the method or approach to engagement. In total, 29.0% (79/272) of the included evidence syntheses were judged to provide few or inadequate details (“red”), 52.2% (142/272) judged to provide a brief or partial description (“amber”), and 18.8% (51/272) judged to provide a comprehensive description of one, or more, method(s) or approach(es) to engagement (“green”).

**Table 7:** Comprehensiveness of description of method or approach to involvement.

Reports of interest‐holder engagement in a single evidence synthesis	Green		Amber		Red		Total
Evidence synthesis	41	18.1%	114	50.4%	71	31.4%	226
Description of methods of engagement	9	75.0%	2	16.7%	1	8.3%	12
Report of a guideline/recommendation	1	2.9%	26	76.5%	7	20.6%	34
Total	51	18.8%	142	52.2%	79	29.0%	272

### 6.2 Evidence Syntheses Providing Comprehensive Description of Engagement (“Green” Evidence Syntheses) (*n* = 51)

Table 8 provides an overview of engagement described within the 51 evidence syntheses categorized as “green.” These comprise 10 quantitative systematic reviews, 8 qualitative and mixed‐method evidence syntheses, 13 scoping reviews, 14 realist reviews, 3 overviews of reviews, and 3 publications that each reported more than one single evidence synthesis. The 51 evidence syntheses were published in 30 unique journals (see Table 9). Narrative summaries, grouped according to type of evidence synthesis (Appendix 7), bring together descriptions of who was engaged, how they were recruited, what happened at what stage of an evidence synthesis, and the level of control that engaged interest‐holders had over the evidence synthesis. Key characteristics are briefly described below.

**Table 8:** Overview of 51 “green” papers.

Study	Type of paper	Stated aim of publication/evidence synthesis	Topic/focus of evidence synthesis	Country	Total number engaged	Patients/public/carers engaged	How were people recruited?
Bakaki et al. [50]	Scoping review	To report the methods of our transdisciplinary scoping review of pediatric polypharmacy. To facilitate potential replication by others, we also describe the key roles of our transdisciplinary team members.	Other: Pediatric polypharmacy	USA	7	0	Closed, invitation
Bennett et al. [63]	Overview of reviews	To create a map of the currently available evidence on patient and family engagement strategies that have been used to help people manage chronic conditions.	Intervention (not a specific population)	USA	11	4	Unclear/not reported
Bergin et al. [64]	Quantitative review	To improve understanding of the nature and impact of patient and public involvement in cancer prevention, screening, and early detection research.	02 Neoplasms	Australia	2	2	Closed, invitation
Browne et al. [71]	Scoping review	To summarize evidence on fidelity and key elements of motivational interviewing‐based interventions for managing adolescent obesity and examine the reporting of these interventions.	05 Endocrine, nutritional, and metabolic diseases	Canada	13	0	Closed, purposive sampling
Brutt et al. [72]	Quantitative review	For patients to contribute and prioritize clinically relevant outcomes to a systematic review on meta‐cognitive interventions, and to evaluate a patient workshop, as well as patients' perceptions of research involvement.	06 Mental, behavioral, or neurodevelopmental disorders	Germany	7	7	Closed, invitation
Bunn et al. [74]	Realist review	To identify key features or mechanisms of programs and approaches that strengthen relationships between community HSCPs, patients with multiple health and care needs and their family carers; and to provide a context‐relevant understanding of how models to facilitate shared decision making might work for older people with multiple health and care needs, and how they might be used to facilitate person‐centered care in collaborative models of health and social care.	Intervention (not a specific population)	UK	24	15	Closed, purposive sampling
Carr et al. [82]	Scoping review	To answer the research question, “what is known from peer reviewed literature about mental health service user experiences of social and psychological harm in social care in England?.”	06 Mental, behavioral, or neurodevelopmental disorders	UK	7	Unclear	Unclear/not reported
Corp et al. [93]	Quantitative review	To identify characteristics associated with self‐directed self‐management interventions that aimed, in whole or part, to address distress, well‐being, or self‐efficacy in young people with physical long‐term conditions.	Other: young people with long‐term conditions	UK	7	7	Closed, existing group
Davies et al. [98]	Realist review	To identify the Program Theory that will inform Assisted Decision Making implementation in healthcare.	Intervention (not a specific population)	Ireland	15	Unclear	Unclear/not reported
de Bell et al. [102]	Overview of reviews	To provide an overview of the volume, diversity, and nature of recent systematic reviews on the effectiveness, acceptability, and implementation of remote monitoring for adults with long‐term physical health conditions.	Intervention (not a specific population)	UK	12	5	Unclear/not reported
Edwards et al. [117]	Realist review	To identify contexts and mechanisms associated with communication tools, patient decision‐aids, and shared decision‐making approaches that influence patient outcomes in patients with advanced cancer.	02 Neoplasms	UK	Unclear	2	Unclear/not reported
Fleming et al. [127]	Realist review	To understand health system legacies of the Great Recession following the 2008 financial crisis, the underlying mechanisms and their theoretical origins, and how these influenced and impacted health system resilience, and its ability to respond to future shocks.	Other: Health system	Ireland	Unclear	2	Unclear/not reported
Gavin et al. [136]	Qualitative/mixed method review	To incorporate and evaluate public and health professional involvement in a mixed‐methods systematic review of occupational therapy for self‐management of rheumatoid arthritis.	15 Diseases of the musculoskeletal system or connective tissue	UK	21	14	Open, fixed
Goodman et al. [146]	Realist review	To provide a theory‐driven explanation of the effectiveness of programs that aim to improve fecal incontinence in people with advanced dementia in care homes.	08 Diseases of the nervous system	UK	Unclear	7	Closed, invitation
Hallett et al. [153]	Qualitative/mixed method review	To describe the spectrum of negative experiences that people identify while they are inpatients in adult mental health services.	06 Mental, behavioral, or neurodevelopmental disorders	UK	6	Unclear	Unclear/not reported
Hannigan et al. [157]	Qualitative/mixed method review	To answer the research question: “What evidence is there relating to the organisation, provision and receipt of care for people with severe mental illness who have an additional diagnosis of advanced incurable cancer and/or end‐stage lung, heart, renal or liver failure and who are likely to die within the next 12 months?.”	Other: End‐of‐life care	UK	14	4	Closed, invitation
Hanson et al. [158]	Quantitative review	To develop evidence‐based recommendations or suggestions that assist clinicians, clinical laboratories, patients, public health authorities, administrators, and policymakers in decisions related to the optimal use of SARS‐CoV‐2 Ag tests in both medical and nonmedical settings.	12 Diseases of the respiratory system	USA	19	0	Unclear/not reported
Hazelton et al. [164]	Quantitative review	To assess the effectiveness of interventions aimed at perceptual disorders after stroke compared to no intervention or control (placebo, standard care, attention control), on measures of performance in activities of daily living.	Other: 08 Diseases of the nervous system	UK	9	5	Closed, existing group
Hempel et al. [167]	Scoping review	To identify and prioritize areas of psychological health that are important and that can be feasibly addressed by a synthesis of the research literature.	06 Mental, behavioral, or neurodevelopmental disorders	USA	Unclear	0	Closed, invitation
Hunt et al. [173]	Quantitative review	To explore the effectiveness of interventions to prevent or treat adolescent depression and/or anxiety by promoting social inclusion.	06 Mental, behavioral, or neurodevelopmental disorders	Uganda, Turkey, Syria, South Africa, and Egypt	13	13	Unclear/not reported
Hyde et al. [175]	Qualitative/mixed method review	To investigate the process and impact of collaborating with members of a patient Research User Group on a systematic review about shared decision making around prescribing analgesia in primary care consultations.	15 Diseases of the musculoskeletal system or connective tissue	UK	5	5	Closed, existing group
John et al. [178]	Realist review	To investigate which changes to practice work best, in what circumstances, and to what extent, to embed an active role for service users' involvement in recovery‐oriented care planning during acute inpatient care.	24 Factors influencing health status or contact with health services	UK	Unclear	Unclear	Closed, invitation
Johnson et al. [179]	Quantitative review	To report the process for incorporating Patient and public involvement into a Health Technology Assessment (HTA) proposal surrounding pelvic organ prolapse.	16 Diseases of the genitourinary system	UK	5	5	Open, flexible
Joseph‐Williams et al. [181]	Realist review	To develop context‐specific program theories that explain why and how patient decision aids are successfully implemented in routine healthcare settings.	Intervention (not a specific population)	Multiple countries (lead author UK)	18	0	Closed, existing group
Karlsson et al. [182]	Quantitative review	To examine how roles between patients, relatives, and researchers in a broad variety of PPIE activities in health research are described in peer‐reviewed papers, and explore what enables these partnerships.	Research methods	Denmark	11	4	Unclear/not reported
Lourida et al. [206]	Paper reporting multiple reviews	To understand the experience of care in hospital for people living with dementia, their carers, and the staff who care for them, and to assess what we know about improving the experience of care.	08 Diseases of the nervous system	UK	19	3	Unclear/not reported
McCarron et al. [217]	Scoping review	To understand the engagement practices of patients who assume roles as partners in health research.	Research methods	Canada	5	5	Closed, existing group
Merner et al. [222]	Qualitative/mixed method review	To synthesize the views and experiences of consumers and health providers of formal partnership approaches that aimed to improve planning, delivery, or evaluation of health services, and to identify best practice principles for formal partnership approaches in health services by understanding consumers' and health providers' views and experiences.	24 Factors influencing health status or contact with health services	Australia	18	6	Closed, invitation
Millar et al. [224]	Realist review	To provide useful intelligence regarding how, why, and in what circumstances different approaches to interorganisational collaboration are effective in improving the performance of NHS provider organizations.	Intervention (not a specific population)	UK	42	8	Closed, purposive sampling
Moody et al. [229]	Qualitative/mixed method review	To conduct a review of literature reporting on primary care for people with multimorbidity that foregrounds patients' perspectives in the design, conduct, analysis, and reporting of the review, as well as in the content.	24 Factors influencing health status or contact with health services	Canada	2	2	Closed, invitation
Muir et al. [236]	Scoping review	To establish the extent of patient and public involvement [PPI] in emergency care research, identify PPI strategies and processes, and assess the quality of reporting on PPI in emergency care research.	Research methods	Australia	1	1	Closed, invitation
Nesbitt et al. [240]	Scoping review	To map how resilience has been conceptualized and operationalized among transition‐age youth with serious mental illness, explore resilience factors and outcomes that have been studied, and recommend areas for future research.	06 Mental, behavioral, or neurodevelopmental disorders	Canada	20	10	Unclear/not reported
Ni She et al. [242]	Realist review	To identify the mechanisms and resources that enable the reciprocal involvement of seldom heard groups in health and social care research.	Research methods	Ireland	Unclear	Unclear	Unclear/not reported
Oravec et al. [246]	Scoping review	To identify patient and caregiver preferences and prioritized outcomes as they relate to perioperative care in cardiac surgery and its life‐long impact.	11 Diseases of the circulatory system	Canada	19	19	Closed, purposive sampling
Price et al. [257]	Realist review	To identify why, how, in what contexts, for whom, and to what extent remediation interventions work for practising doctors to restore patient safety.	Intervention (not a specific population)	UK	15	2	Unclear/not reported
Reeve et al. [262]	Paper reporting multiple reviews	To review the literature on stopping medicines in older people with multimorbidity and polypharmacy to describe what is being done, where, and for what effect; to construct a program theory that describes “best practice” and helps explain the heterogeneity of deprescribing approaches; and to translate findings into resources to support tailored prescribing in clinical practice.	Intervention (not a specific population)	UK	Unclear	Unclear	Mixed
Rycroft‐Malone et al. [266]	Realist review	To answer the question “how can workforce development interventions improve skills and care standards of support workers within older people's health and social care services?.”	Intervention (not a specific population)	UK	Unclear	Unclear	Closed, purposive sampling
Sanderson et al. [268]	Qualitative/mixed method review	To describe how an integrated knowledge translation approach was embedded within a master's thesis project comprising a mixed‐methods systematic review.	06 Mental, behavioral, or neurodevelopmental disorders	Canada	11	1	Closed, invitation
Schlief et al. [270]	Realist review	To answer the question of what telemental health approaches work, for whom, in which contexts, and how.	06 Mental, behavioral, or neurodevelopmental disorders	UK	28	7	Unclear/not reported
Soobiah et al. [282]	Quantitative review	To quantify the perceived level of engagement experienced by knowledge users involved in different activities in the conduct of a systematic review on the comparative effectiveness of geriatrician‐led models of care across health care settings.	Intervention (not a specific population)	Canada	15	3	Closed, invitation
Todhunter‐Brown et al. [296]	Overview of reviews	To summarize Cochrane Reviews that assessed the effects of conservative interventions for treating urinary incontinence in women.	16 Diseases of the genitourinary system	UK	14	Unclear	Closed, purposive sampling
Troya et al. [300]	Quantitative review	To critically reflect on the process, potential impact, and identify challenges/opportunities in involving robust Patient Public Involvement and Engagement in a doctoral research, including a systematic review and qualitative study.	06 Mental, behavioral, or neurodevelopmental disorders	UK	3	3	Closed, existing group
Turner et al. [302]	Scoping review	To understand how processes at different levels influence the use of evidence in decision‐making on health care innovations.	Other: Innovations in health care	UK	18	5	Closed, invitation
Walker et al. [313]	Paper reporting multiple reviews	To describe and reflect on the methods and influence of involvement of young people with lived experience within a complex evidence synthesis.	06 Mental, behavioral, or neurodevelopmental disorders	UK	8	8	Closed, invitation
Walsh et al. [315]	Scoping review	To explore the benefits, risks, barriers, and enablers for using social media as a tool for stakeholder engagement in health service design and/or quality improvement.	24 Factors influencing health status or contact with health services	Australia	6	3	Closed, purposive sampling
Wang et al. [317]	Scoping review	To answer the questions: (i) what activities have youth with neurodisabilities and their families been engaged in as part of evidence syntheses, and (ii) what were the outcomes of that engagement?	Research methods	Canada	4	2	Closed, existing group
Watson et al. [320]	Scoping review	To identify the psychoeducational interventions utilized with people with complex communication needs, any adaptations to improve communication access, and outcomes for this population.	Intervention (not a specific population)	Australia	6	4	Open, fixed
Welch et al. [323]	Qualitative/mixed method review	To understand the personal and contextual influences of how social self‐management support practices are selected and established in the everyday lives of people with COPD.	12 Diseases of the respiratory system	UK	6	6	Closed, existing group
Zarshenas et al. [329]	Scoping review	To conduct a scoping review of existing lay summary [LS] guidance specific to recommended LS characteristics (i.e., what LSs should look like) and writing processes (i.e., how best to write an LS).	Research methods	Canada	16	12	Closed, purposive sampling
Zhao et al. [331]	Realist review	To understand how and under what circumstances decision coaching works for people making healthcare decisions.	24 Factors influencing health status or contact with health services	Australia, Canada, China, Denmark, Germany, Japan, Norway	8	1	Unclear/not reported
Zibrowski et al. [332]	Realist review	To develop a theory regarding how academic researchers support and sustain patient engagement in patient‐oriented research.	Research methods	Canada	12	3	Unclear/not reported

**Table 9:** Journals in which “green” evidence syntheses were published.

Journal	Journal information relating to engagement	Number of “green” evidence syntheses
*Health Expectations*	Publishes: “about all aspects of patient and public involvement and engagement (PPIE) in health and social care, policy and practice, health and social care research and education of health and social care professionals.” Mandatory reporting of “patient or public contribution.”	9
*NIHR Journals Library*	Mandatory reporting of PPI.	8
*Research Involvement and Engagement*	Publishes: “on patient and public involvement and engagement in health and social care research.” Use of GRIPP2 encouraged.	3
*Cochrane Database of Systematic Reviews*	Cochrane supports “consumer involvement throughout the entire process of research and dissemination,” but no mandatory reporting prior to 2025.	3
*International Journal of Environmental Research and Public Health*	No relevant information identified	2
*International Journal of Health Policy and Management*	No relevant information identified	2
*Agency for Healthcare Research and Quality*	No relevant information identified	1
*Alzheimer's and Dementia*	No relevant information identified	1
*BMC Health Services Research*	No relevant information identified	1
*BMC Medical Informatics and Decision Making*	No relevant information identified	1
*BMC Medical Research Methodology*	No relevant information identified	1
*BMC Psychiatry*	No relevant information identified	1
*British Journal of Occupational Therapy*	No relevant information identified	1
*British Journal of Social Work*	No relevant information identified	1
*Child: Care, Health and Development*	No relevant information identified	1
*Clinical Infectious Diseases*	No relevant information identified	1
*Disability and Rehabilitation*	No relevant information identified	1
*Dissertation Abstracts International*	No relevant information identified	1
*Emergency Medicine Journal*	No relevant information identified	1
*Implementation Science*	No relevant information identified	1
*Interactive Journal of Medical Research*	No relevant information identified	1
*International Journal of Nursing Studies*	No relevant information identified	1
*International Journal of Technology Assessment in Health Care*	No relevant information identified	1
*Journal of Clinical Epidemiology*	No relevant information identified	1
*Journal of Thoracic and Cardiovascular Surgery*	No relevant information identified	1
*Medical Care*	No relevant information identified	1
*Medical Decision Making*	No relevant information identified	1
*Obesity Reviews*	No relevant information identified	1
*Preventive Medicine*	No relevant information identified	1
*Zeitschrift fur Evidenz, Fortbildung und Qualitat im Gesundheitswesen (Journal for Evidence, Continuing Education and Quality in Healthcare)*	No relevant information identified	1
*Total unique journals*	*2 mandate reporting of interest‐holder engagement; 2 encourage/support interest‐holder engagement; 26 have no information relating to interest‐holder engagement*.	*51 publications in 30 unique journals*

#### 6.2.1 Interest‐Holders Engaged

Patients, caregivers, family members, representatives of patient/caregiver organizations, or general members of the public (abbreviated to “patients/public” below) were engaged in most of the evidence syntheses (43/51, 84.3%). For over half (30/51, 58.8%) of the “green” evidence syntheses, other interest‐holders (professionals) were engaged alongside the patients/public members, although around one‐quarter (13/51, 25.5%) only engaged patients/public members. For a small number (6/51, 11.8%), there were only professional interest‐holders engaged. For two of the “green” evidence syntheses, patients/caregivers were engaged, but it was unclear if there were also professionals engaged. Table 10 summarizes the types of interest‐holders engaged and provides publication references.

**Table 10:** Summary of interest‐holders engaged in “green” evidence syntheses.

Interest‐holders engaged	“Green” papers		References
Patients/carers/family/public only	13	25.5%	[64, 72, 82, 93, 173, 179, 217, 229, 236, 246, 300, 313, 315]
Patients/carers/family/public + other interest‐holders (professionals)	30	58.8%	[63, 74, 98, 102, 117, 127, 136, 146, 157, 164, 178, 206, 222, 224, 240, 242, 257, 266, 268, 282, 296, 302, 315, 317, 320, 329, 331, 332]
Professional interest‐holders only	6	11.8%	[50, 71, 98, 158, 167, 181]
Patients/carers/family/public, but unclear if other interest‐holders (professionals)	2	3.9%	[153, 182]
Total	51	100%	

We attempted to categorize the type of interest‐holder using the 11 “P's” [7] (see Box 1). Over two‐thirds of “green” evidence syntheses (68.6%, 35/51) provided some information about the type of professional interest‐holders. Of those reporting this information, most commonly the professional interest‐holders were “providers” (95.3%, 33/35), but engagement of interest‐holders who were researchers (“principal investigators”) was also common (60.0%, 21/35). The engagement of producers/commissioners, program managers, and policy makers was reported in 22.9% (8/35), 20.0% (7/35), and 8.6% (3/35) of “green” evidence syntheses, respectively, whilst one evidence synthesis clearly reported the engagement of a payer of health research, and none reported engagement of peer‐reviewed journal editors. Several “other” interest‐holders, whom it was challenging to categorize using the 11 Ps, were reported to be engaged; these included people involved in education and law, care home managers, administrators, and students.

#### 6.2.2 Aim of Engagement

The Table of Included studies (Supporting Information S1) provides the author description of engagement, including, where provided, the aim or goal of engagement. Generally, the aim of engagement was articulated in relation to the roles and activities that the interest‐holders were engaged in, and to the stage in the evidence synthesis process, and it was challenging to synthesize these in relation to the aim of engagement. Commonly, broad terms and phrases were used, for example: “to gain feedback” [102], “to help define the scope” [117], “to interpret” [175], “to get an expert opinion” [178], “to discuss findings” [222]. Several “green” evidence syntheses referred to the goal of including the voice or perspectives of the interest‐holders, for example: “informed by both patient and healthcare professional perspectives” [236]; “to ensure patient voice is heard throughout the review” [153]. Although an overall aim was often not provided, aims were commonly stated in relation to the tasks or activities that the interest‐holders were engaged in, for example: “to comment on preliminary themes, and guide final analysis, to identify areas not covered by the literature)” [153], “to ensure that our findings were relevant to the people who would eventually use them” [206], “prioritize clinically relevant outcomes” [72].

#### 6.2.3 Characteristics of Interest‐Holders Engaged

We explored whether the demographic characteristics of the interest‐holders engaged were reported according to the PROGRESS‐Plus domains [25]. Half of the “green” evidence syntheses (51.0%, 26/51) did not report any characteristics of the interest‐holders engaged, and none of them reported any information relating to domains of religion, socioeconomic status, or social capital. Over a quarter (27.5%, 14/51) provided some information about gender, 23.5% (12/51) about age and 21.6% (11/51) about place of residence. One‐fifth (19.6%, 10/51) reported occupation, but this often related to the professional interest‐holders. Only 9.8% (5/51) reported any information about race/ethnicity/culture, 9.8% (5/51) about disability and 5.9% (3/51) about education.

#### 6.2.4 How Interest‐Holders Were Invited to be Engaged

In one‐third of “green” evidence syntheses (17/51, 33.3%) it was not clearly reported how interest‐holders were recruited. In 58.8% (30/51) there was a “closed” recruitment strategy (see Box 1). The membership of the closed group was formed by individually inviting interest‐holders to the group (14/51, 27.5%), engaging members of an existing group (8/51, 15.7%), or using a purposeful sampling approach (8/51, 15.7%). The recruitment approach was only considered to be “open” in 3/51 (5.9%), with opportunities for any interest‐holder(s) to volunteer to contribute. One “green” evidence synthesis used multiple recruitment approaches, comprising both open and closed strategies, for engaging interest‐holders in different parts of their process.

#### 6.2.5 Geographical Location of Interest‐Holders Engaged

The countries in which engagement was conducted are reported in Table 8. In most, these were European (28/51, 54.9%) or North American (15/51, 29.4%) countries, with few in East Asian/Pacific countries (5/51, 9.8%). In three evidence syntheses (3/51, 5.9%), engagement occurred across several different countries.

#### 6.2.6 Ethical Approval

Almost half of the “green” evidence syntheses (24/51, 47.1%) did not report any information relating to ethics or ethical approval. A quarter (13/51, 25.5%) reported that ethical approval was sought and approved, and 17.3% (14/81) reported that ethical approval was not required. Almost all (12/13, 92.3%) “green” evidence syntheses that sought ethical approval involved patients, carers, and/or family members, while the type of interest‐holder was unclear for the remaining evidence synthesis.

#### 6.2.7 Compensation

Table 11 summarizes information relating to payment and other forms of compensation given to interest‐holders. Over three‐quarters of “green” evidence syntheses did not report whether expenses were paid to engaged interest‐holders (40/51, 78.4%), with less than one‐fifth clearly reporting that out‐of‐pocket expenses were paid (9/51, 17.6%), and two evidence syntheses (2/51, 3.9%) explicitly stating that interest‐holders were not paid. Similar proportions report information relating to other forms of compensation (including financial payments, vouchers, or other rewards provided to interest‐holders), with three‐quarters not providing any information (39/51, 76.5%), one‐fifth reporting that some form of compensation was provided (11/51, 21.5%), and one stating that no compensation was provided (1/51, 2.0%).

**Table 11:** Summary of information relating to compensation provided to interest‐holders reported in 51 “green” papers.

	Yes		No – states not paid		Not reported	
Were expenses paid? (i.e., out‐of‐pocket expenses such as travel, childcare, or internet access)	9	17.6%	2	3.9%	40	78.4%
Were people engaged, given any other compensation (financial payment, voucher, other reward)	11	21.5%	1	2.0%	39	76.5%

#### 6.2.8 Tool or Method of Reporting Engagement

Twelve percent of “green” evidence syntheses (6/51, 11.8%) used or referred to the ACTIVE Framework, 21.6% (11/51) used the GRIPP/GRIPP2 checklist. Other checklists/tools used included the Concannon et al.'s 7Ps framework [335], “Linkage and exchange model” [336], UK Standards for Public Involvement [13], Saskatchewan Center for Patient‐Oriented Research level of engagement tool (PORLET) [337], CIHR Guiding Principles for Patient Engagement Framework [338], an Involvement Matrix [339], and a range of tools specific to evidence synthesis methodology (e.g., RAMESES [340]; COREQ [341]).

#### 6.2.9 When Was There Engagement?

Table 12 summarizes at what stage in the evidence synthesis process there were reported engagement activities within each of the “green” evidence syntheses. Engagement was most frequently reported at the stage of interpretation of findings (39/51, 76.5%). Next most frequent stage at which engagement was reported was during the data synthesis stage (9. Analyze/synthesize data; 31/51, 60.8%) and at the beginning of the evidence synthesis (1. Develop question (26/51, 51.0%) and 2. Plan methods (21/51, 41.2%)). It was common for interest‐holders to be engaged at both the beginning and end stages of an evidence synthesis, that is, using a “top and tail” approach [5]. In addition to the activities reported at specific evidence synthesis stages, around one‐third of the evidence syntheses described one or more interest‐holders who were engaged “throughout,” but without detailing specific activities.

**Table 12:** Summary of when the ES engagement was reported to occur within the 51 “green” papers.

Key stages of an ES	Volume of “Green” papers (*n* = 51)	
Before the review	11	21.6%
1. Develop question	26	51.0%
2. Plan methods	21	41.2%
3. Protocol	7	13.7%
4. Develop search	15	29.4%
5. Run search	5	9.8%
6. Select studies	12	23.5%
7. Collect data	12	23.5%
8. Assess ROB	3	5.9%
9. Analyze/synthesize data	31	60.8%
10. Interpret findings	39	76.5%
11. Write and publish a review	20	39.2%
12. Knowledge translation	19	37.3%
Throughout the ES	11	21.6%

*Note:* The darker the green shade, the greater the proportion of ES reporting engagement at that stage of an ES.

Appendix 8 provides a more detailed synopsis of the stages in the evidence synthesis process at which there was engagement. This detailed breakdown of activities shows that there was engagement reported within every activity, which may be part of an evidence synthesis, although only one “green” evidence synthesis specifically described engagement during the activity of “run search.”

#### 6.2.10 Reflexivity

We judged that one‐third of “green” publications (31.4%, 16/51) reported on their interest‐holder engagement using a process of reflexivity, providing a reflective discussion relating to the engagement activities that had been undertaken during the evidence synthesis. All evidence syntheses using the GRIPP2 reporting tool [20] were considered to provide some reflexivity.

#### 6.2.11 Conflicts of Interest

Almost all “green” evidence syntheses (96.1%, 49/51) reported conflicts of interest of the authors, but only one‐third (33.3%, 17/51) reported any conflicts of interest of the engaged interest‐holders. Where conflicts of interest of interest‐holders engaged were reported, this generally occurred when all of those engaged were co‐authors.

#### 6.2.12 Co‐Authorship

In over half (52.3%, 27/51) of the “green” evidence syntheses, at least one interest‐holder was a co‐author. The role and responsibilities of a co‐author varied considerably between different evidence syntheses; some co‐authors worked as equal members of a review team, and other co‐authors contributed to specific, and often limited, tasks only (e.g., commenting on written drafts of manuscripts). Often, in addition to having one or two patient/public co‐authors, there were additional interest‐holders engaged in specific tasks throughout the evidence synthesis process.

#### 6.2.13 How Were Interest‐Holders Engaged?

Supporting Information S2 provides an overview of the different forms of engagement, methods used to facilitate engagement, and the level of control that interest‐holders were judged to have during the different stages/activities at which there was engagement in the 51 “green” evidence syntheses. A brief summary of how interest‐holders were engaged in the most common types of evidence syntheses is provided here (for more details, see Appendix 7):

- Quantitative systematic reviews (*n* = 10): In four evidence syntheses, interest‐holders were involved in multiple activities throughout the review process [64, 158, 182, 300]; in three, engagement occurred during the early stages [72, 179, 282]; and three adopted a “top and tail” approach [93, 164, 173]. Commonly, activities comprised meetings (both face‐to‐face and online) and email communication, but other activities included larger workshops, ranking of outcomes, and surveys.
- Qualitative and mixed method evidence syntheses (*n* = 8): Five of the eight evidence syntheses reported that they had continuous engagement throughout, with four of these also having other engagement activities for specific evidence synthesis tasks [136, 153, 222, 268], while one had continuous involvement of two patient partners [229]. Two used a “top and tail” approach [157, 175], while one had engagement only during the final evidence synthesis stages [323]. None of these evidence syntheses reported the use of any formal research methods or techniques to engage people, with “meetings” being the most common form of engagement.
- Scoping reviews (*n* = 13): Eight of the 13 scoping reviews had engagement throughout the review [50, 82, 217, 236, 246, 315, 317, 329]; three had engagement during the final stages only [71, 302, 320], one adopted a “top and tail” approach [240], and one engaged interest‐holders in rating potential evidence synthesis topics [167]. Five evidence syntheses used meetings or a mixture of meetings and electronic communication to engage interest‐holders [217, 246, 315, 317, 329]. Four evidence syntheses included interviews or focus groups [71, 240, 302, 320] and two used electronic communication only [50, 82].
- Realist reviews (*n* = 14): All the included realist reviews reported engagement with topic experts and/or patient and public representatives to generate initial program theories [74, 98, 117, 127, 146, 178, 181, 224, 242, 257, 266, 270, 331, 332]. The other main activity in which people were engaged was to refine and validate the final program theory. Engagement in this activity was reported in all the realist reviews. Interest‐holders were often asked to provide feedback on the preliminary analysis (or Context‐mechanism‐outcome configurations (CMOCs) in realist reviews) and to test or refine the program theories that were developed from the analysis. This was done primarily through interviews, workshops, and team meetings.

Across all types of evidence synthesis, most commonly, interest‐holders attended meetings at which no formal methods of engagement were used. Often, attendance at meetings was supplemented with electronic communication, such as emails. However, several “green” evidence syntheses described group discussion, sometimes referred to using the term “consultation.” Formal consensus activities, such as voting or ranking techniques, or the use of nominal group technique or Delphi surveys, were reported by some, while others reported holding larger “workshops,” “focus groups,” or individual interviews. It was most commonly considered that the interest‐holders had some influence over decisions and outcomes of each activity, though there were a small number of evidence syntheses in which it was judged that the interest‐holders had overall control over final decisions (possibly sharing equal power with the research team). With the exception of the engagement in activities relating to the generation, refinement, and validation of program theories within realist reviews, we were unable to identify any obvious patterns in relation to different forms of engagement, methods used, or level of control during different stages of an evidence synthesis. Across all types of evidence syntheses, it was common to engage interest‐holders in multiple activities throughout the review process, but there are also examples where interest‐holders are engaged for one activity only. Where interest‐holders contributed to one activity only, this is most likely to be an interpretation of review findings.

### 6.3 Publications Providing an Account of Description of Engagement, or Methods of Engagement, Relevant to Evidence Syntheses (“Methods” Papers; *n* = 44)

The included “methods” papers (44/302, 14.6%) comprised an eclectic mix of publications, but have been broadly categorized into publications which:

- Describe a method or methods of engagement, or guidance relating to how to engage interest‐holders (*n* = 17)
- Focus on a specific methodological approach, tool, or framework (*n* = 9)
- Evaluate or reflect on engagement (*n* = 7)
- Describe methods to identify and/or prioritize new reviews (*n* = 7)
- Focus on review publication and dissemination (*n* = 2)
- Address other topics (*n* = 2)

Details of these publications are provided in Table 13.

**Table 13:** Summary of other publications providing relevant information relating to engagement in evidence syntheses (“Methods” papers; *n* = 44).

Paper	Title	Stated aim/objective	Key notes
*Describes methods of engagement/provides guidance relating to how to engage interest‐holders*			
Abrams et al. [30]	Lost in reviews: Looking for the involvement of stakeholders, patients, public, and other non‐researcher contributors in realist reviews.	To (i) describe the ways in which contributors have been involved in realist reviews, with a particular focus on PPI, and (ii) document how involvement has been reported.	Describes how interest holders have been involved in **realist reviews**.
Arntsen et al. [41]	Patient‐centered health technology assessment: A perspective on engagement in health technology assessment by three patient organizations and a health technology assessment body.	To analyze the experience of being involved in an Institute for Clinical and Economic Review (ICER) HTA review in the United States.	Describes experiences of involvement in HTA reviews. Brings perspectives of three patient organizations.
Buus et al. [79]	Arksey and O'Malley's consultation exercise in scoping reviews: A critical review.	To explore how consultation exercises were described in a convenience sample of recent scoping reviews.	A scoping review, focussed on engagement of interest‐holders in **scoping reviews**.
Dewidar et al. [108]	Methodological guidance for incorporating equity when informing rapid‐policy and guideline development.	To provide guidance on incorporating equity throughout the rapid review process and provide examples from published COVID‐19 rapid reviews to illustrate its application.	Guidance focussed on how to involve interest‐holders in **rapid reviews**.
Garritty et al. [134]	Rapid reviews methods series: Involving patient and public partners, healthcare providers, and policymakers as knowledge users.	To discuss the importance of knowledge users' involvement and to highlight potential ways to engage users and detail stages of involvement in the rapid review process.	Describes how interest holders have been involved in **rapid reviews**.
Haddaway et al. [152]	A framework for stakeholder engagement during systematic reviews and maps in environmental management.	To provide a toolbox of possible stakeholder engagement activities, whilst also recommending approaches from stakeholder engagement research that may prove to be particularly useful for reviews.	Development of a framework and “toolbox” of interest‐holder activities.
Helmer et al. [166]	Dissemination of knowledge from Cochrane Public Health reviews: A bibliographic study.	To identify (i) dissemination strategies and (ii) stakeholders of Cochrane Public Health reviews.	Describes the engagement of interest‐holders in Cochrane Public Health reviews, with a focus on dissemination.
Karlsson et al. [182]	Roles, outcomes, and enablers within research partnerships: A rapid review of the literature on patient and public involvement and engagement in health research.	To examine how roles between patients, relatives, and researchers in a broad variety of PPIE activities in health research are described in peer‐reviewed papers, and explore what enables these partnerships.	Investigates the roles, outcomes, and enablers of involvement in health research, including evidence syntheses.
Langer et al. [193]	How stakeholder engagement has led us to reconsider definitions of rigour in systematic reviews.	To address head‐on the often undiscussed key challenge with regard to stakeholder involvement in systematic reviews: That responding to stakeholders can mean reconsidering what makes a review rigorous. It proposes a new model to address these tensions that combines the production of public good reviews with stakeholder‐driven syntheses.	Provides a commentary on stakeholder involvement in SRs. Proposes a “new model” for stakeholder involvement in systematic reviews.
Mann et al. [211]	Palliative Care Evidence Review Service (PaCERS): A knowledge transfer partnership.	To describe Palliative Care Evidence Review Service (PaCERS), a methodology utilizes modified systematic review methods, as there is no agreed definition or accepted methodology for conducting rapid reviews. This paper describes the stages involved based on our iterative recent experiences and engagement with stakeholders, who are the potential beneficiaries of the research.	Describes a detailed approach to conducting **rapid reviews**, including stakeholder involvement.
Morley et al. [232]	A systematic scoping review of the evidence for consumer involvement in organisations undertaking systematic reviews: Focus on Cochrane.	To conduct a systematic scoping exercise to evaluate the evidence base on consumer involvement in organizations that commission, undertake, or support systematic reviews, with an emphasis on Cochrane.	Explores engagement within organizations involved in conducting evidence syntheses.
Petticrew et al. [252]	The Commercial Determinants of Health and Evidence Synthesis (CODES): Methodological guidance for systematic reviews and other evidence syntheses.	To provide guidance on the conduct of systematic reviews focussed on Commercial Determinants of Health [CODH], from shaping the review question with input from stakeholders, to disseminating the review.	Development of guidance relating to systematic review conduct, including interest‐holder engagement.
Pollock et al. [12]	Stakeholder involvement in systematic reviews: A scoping review.	To document the evidence base relating to stakeholder involvement in systematic reviews and use this evidence to describe key features of how stakeholders have been involved in systematic reviews.	This is the earlier version of this current scoping review.
Pollock et al. [254]	Moving from consultation to co‐creation with knowledge users in scoping reviews: Guidance from the JBI Scoping Review Methodology Group.	To provide a pragmatic how‐to guide to help encourage scoping reviewers to include knowledge users within the conduct and reporting of scoping reviews.	A guide focussed on how to involve interest‐holders in **scoping reviews**.
Sakala et al. [267]	A consumer viewpoint. Consumer‐professional partnership to improve research: The experience of the Cochrane Collaboration's Pregnancy and Childbirth Group.	To describe an innovative pilot project to involve consumers and consumer advocates in the process of refereeing systematic reviews in preparation by members of the Cochrane Collaboration's Pregnancy and Childbirth Group.	Describes involvement of consumers in Cochrane Reviews (providing feedback on protocols and reviews).
Tricco et al. [299]	Engaging policy‐makers, health system managers, and policy analysts in the knowledge synthesis process: A scoping review.	To map the literature on engaging knowledge users in the knowledge synthesis process.	A scoping review, focussed on engagement of policy makers, health systems managers, and policy analysts in evidence synthesis.
Wang et al. [317]	Youth and family engagement in childhood disability evidence syntheses: A scoping review.	To answer (i) what activities have youth with neurodisabilities and their families been engaged in as part of evidence syntheses, and (ii) what were the outcomes of that engagement?	Explores the involvement of youth and families in disease‐specific evidence syntheses.
*Specific methodological approach*			
Arnstein et al. [40]	Patient involvement in preparing health research peer‐reviewed publications or results summaries: A systematic review and evidence‐based recommendations.	(1) conduct a systematic review of the evidence on patient involvement in results sharing, (2) propose evidence‐based recommendations to help maximize benefits and minimize risks of such involvement, and (3) conduct this project with patient authors.	Patient Authorship Experience tool.
Beames et al. [62]	A new normal: Integrating lived experience into scientific data syntheses.	To describe in an opinion piece how lived experiences can be integrated into evidence synthesis.	Describes a way of integrating lived experiences within evidence synthesis.
Cooper et al. [90]	Blurring the boundaries between synthesis and evaluation. A customized realist evaluative synthesis into adolescent risk behavior prevention.	To set out the challenges faced in conducting the review, outline the steps taken to customize the realist methodology, and discuss how this customized methodology was used to overcome these challenges. This study aims to contribute to the realist methodological literature, to provide an example of methodological customization, and to consider the potential usefulness of using an evaluative synthesis approach in conducting future realist research.	Describes the development of a customized **realist** evaluative synthesis, incorporating interest‐holder expertise within theory refinement, and also combining it with the VICTORE checklist.
Dion et al. [109]	Weight of evidence: Participatory methods and Bayesian updating to contextualize evidence synthesis in stakeholders' knowledge.	To present the approach “Weight of Evidence” as a transformative procedure for stakeholders to interpret, expand on, and prioritize evidence from evidence syntheses, with a focus on engaging populations historically excluded from planning and decision making.	Weight of evidence method.
Gould et al. [150]	Systematic review and knowledge translation: A framework for synthesizing heterogeneous research evidence.	To consider how knowledge translation strategies can support and advance systematic reviews that include diverse types of research. Lessons learned from conducting a systematic review of Americans with Disabilities Act (ADA) employment research are explained and contextualized within research on barriers and facilitators to successful knowledge translation.	Describes how interest‐holders gave feedback to enhance the validity and confirmability of a knowledge translation model.
Land et al. [192]	A five‐step approach for stakeholder engagement in prioritisation and planning of environmental evidence syntheses Neal Haddaway, Sally Crowe.	To report on the empirically tested five‐step approach as used by the Mistra Council for Evidence‐based Environmental Management (EviEM). This approach describes how to engage stakeholders and incorporate their views and opinions in the prioritization and planning of reviews.	Five‐step approach for stakeholder engagement in prioritization and planning of environmental evidence syntheses.
Martinez et al. [214]	Stakeholder engagement in research: A scoping review of current evaluation methods.	To describe approaches for evaluation of stakeholder engagement rather than to appraise evaluation rigor or the engagement methods used. In this paper, we present a descriptive overview of our findings and identify areas for future research toward inclusive and systematic evaluations of stakeholder engagement.	Methods of evaluating engagement in research (including, but not limited to, evidence syntheses).
Munthe‐Kaas et al. [237]	User experiences of structured stakeholder engagement to consider transferability: The TRANSFER approach.	We aim to explore the user experience of the TRANSFER approach conversation guide, and in doing so, gain a better understanding of the role and perceived value of stakeholder engagement in systematic reviews for informed decision‐making. (The TRANSFER approach is a novel method that aims to support review authors to systematically and transparently collaborate with stakeholders to consider context and the transferability of review findings from the beginning of the review process).	TRANSFER approach conversation guide.
Pollock et al. [5]	Development of the ACTIVE framework to describe stakeholder involvement in systematic reviews.	To report the development of the ACTIVE framework to describe how stakeholders are involved in systematic reviews.	ACTIVE Framework.
*Evaluation/reflection on engagement*			
Agyei‐Manu et al. [34]	The benefits, challenges, and best practice for patient and public involvement in evidence synthesis: A systematic review and thematic synthesis.	To synthesize qualitative evidence on the benefits, challenges, and best practices for PPI in ES/SR projects from the perspectives of patients/public and researchers.	A qualitative review, bringing together evidence about the benefits and challenges of PPI for evidence synthesis projects.
Aschmann et al. [42]	Informing patient‐centered care through stakeholder engagement and highly stratified quantitative benefit‐harm assessments.	To discuss systematically engaging diverse stakeholders and stratification in quantitative benefit‐harm assessments, and consider feasibility and usefulness for the process of guideline development.	Describes a process of engagement of interest‐holders in “benefit‐harm assessments,” based on synthesized evidence, to inform guideline development.
Bayliss et al. [61]	Patient involvement in a qualitative meta‐synthesis: Lessons learnt.	To inform the evidence base on effective ways of involving patients in a qualitative meta‐synthesis.	Reports findings from a questionnaire sent to eight patient research partners who had contributed to a **qualitative evidence synthesis**.
Boden et al. [67]	Patient partners' perspectives of meaningful engagement in synthesis reviews: A patient‐oriented rapid review.	To investigate how research teams can ensure that patient partner (PP) contributions to synthesis reviews (SRs) are meaningful from the PPs perspective.	A qualitative review, bringing together patient partner reflections on engagement in evidence synthesis.
Cornman et al. [92]	Discreting the perception and impact of patient involved in evidence‐based practice center key informant interviews.	To examine how patients, caregivers, and patient advocates who participated as Key Informants in prior systematic reviews regarded that experience, and what their recommendations are for improving that process.	Reports findings from interviews with caregivers and patient advocates who participated as “key informants” for a systematic review.
Gierisch et al. [142]	Qualitative exploration of engaging patients as advisors in a program of evidence synthesis: Cobuilding the science to enhance impact.	To explore views, barriers, resources, and perceived values of engaging patient advisors in a national program of evidence synthesis research.	Reports findings from interviews with patient interest‐holders who contributed to an evidence synthesis program.
Merner et al. [221]	Stakeholder involvement in systematic reviews: Lessons from Cochrane's Public Health and Health Systems Network.	To present the lessons of stakeholder involvement experiences from four major research activities in systematic review prioritization, production, and dissemination.	Reports four case‐studies, with the researcher's reflections on the interest‐holder engagement.
*Methods to identify and prioritize new reviews*			
Anderson et al. [38]	Synthesis for health services and policy: Case studies in the scoping of reviews.	To describe and discuss the experiences of review scoping of three commissioned research centers that conducted evidence syntheses to inform health and social care organization, delivery, and policy in the United Kingdom, between 2017 and 2020.	Identifies key issues in the early (scoping) phase of evidence syntheses.
Dennett et al. [107]	A survey and stakeholder group prioritised key systematic review questions in airways disease.	To (i) prioritize 10 reviews of importance to the public (patients, carers, healthcare professionals, and researchers) from a patient survey, (ii) to engage stakeholders with expertise and lived experience across our scope in our priority setting processes, and (iii) to identify potential new stakeholders, contributors, and funding streams.	Cochrane Airways Group priority setting project.
O'Connor et al. [243]	A rapid priority setting exercise combining existing, emergent evidence with stakeholder knowledge identified broad topic uncertainties.	To engage with key international stakeholders to rapidly identify topic uncertainties, rank them, and gather broad insight on those deemed most important.	Cochrane Airways Group priority setting project.
Scott et al. [271]	Cochrane Acute Respiratory Infections Group's stakeholder engagement project identified systematic review priority areas.	To report the results of a prioritization project, aiming to identify the highest priority systematic review topics.	Cochrane Acute Respiratory Infection priority setting project.
Sigfrid et al. [276]	A rapid research needs appraisal methodology to identify evidence gaps to inform clinical research priorities in response to outbreaks—Results from the Lassa fever pilot.	To develop and pilot a protocol for carrying out a systematic, rapid research needs appraisal (RRNA) of existing evidence within 5 days in response to disease outbreaks.	Describes the development of the Rapid Research Needs Appraisal (RRNA) and piloting with interest‐holders.
Synnot et al. [288]	Evaluation of the Cochrane Consumers and Communication Group's systematic review priority‐setting project.	To describe the evaluation of the priority setting project for systematic reviews in partnership with stakeholders (consumers/patients, health professionals, policy‐makers, and others).	Cochrane Consumers and Communication Group priority setting project.
Tomlinson et al. [298]	Prioritising Cochrane reviews to be updated with health equity focus.	To prioritize Cochrane reviews of interventions to be updated with a health equity lens, where it is important to understand the distribution of effects across one or more PROGRESS‐Plus characteristics.	Cochrane Health Equity reviews prioritization project.
*Review publication and dissemination*			
Heenan et al. [165]	Combining public health evidence, policy experience and communications expertise to inform preventive health: Reflections on a novel method of knowledge synthesis.	We describe and outline the rationale for this policy‐relevant knowledge synthesis approach, the theories that informed its development, and present two case studies of how the process was used and adapted in practice.	Describes a policy‐relevant knowledge synthesis approach.
Zarshenas et al. [329]	Recommended characteristics and processes for writing lay summaries of healthcare evidence: A co‐created scoping review and consultation exercise.	To conduct a scoping review of existing lay summary [LS] guidance specific to recommended LS characteristics (i.e., what LSs should look like) and writing processes (i.e., how best to write an LS).	Focus on lay summary guidance.
*Other*			
Eales et al. [114]	Much at stake: The importance of training and capacity building for stakeholder engagement in evidence synthesis.	To identify five broad categories of training across evidence synthesis processes, from question formulation to communication of findings, where training is important for effective two‐way communication among the full range of different stakeholder groups.	Discusses training issues for involving interest‐holders in evidence syntheses.
Masterson et al. [342]	Mapping definitions of co‐production and co‐design in health and social care: A systematic scoping review providing lessons for the future.	This study aimed to explore how the concepts of co‐production and co‐design have been defined and applied in the context of health and social care and to identify the temporal adoption of the terms.	Maps concepts and definitions of co‐production in existing literature.

## 7 Comparison With Results of Previous Review

Our previous scoping review [15] included 291 publications; our updated search has identified a further 302 publications. Details of the 291 publications are provided in the supplementary material to the previous scoping review paper [15]. Tables summarizing the results from both the updated review and the previous review are provided in Supporting Information S3.

## 8 Discussion

### 8.1 Summary of Findings

This scoping review has collated and provided an overview of 302 publications that describe the engagement of interest‐holders in a single evidence synthesis. We have described key details relating to the engagement in 51 evidence syntheses, which were judged to provide the most comprehensive description of engagement. Box 2 provides an overview of the findings of this scoping review, in relation to each of the pre‐stated review objectives.

Box 2 **Overview of the findings of this scoping review, in relation to each of the pre‐stated review objectives.**

**Overview of key findings in relation to each pre‐stated review objective**

**Objective 1. Identify and collate resources that describe the engagement of interest‐holders in evidence syntheses**

We identified 302 publications, published since the previous scoping review, which describe the engagement of interest‐holders in a single evidence synthesis. Most publications (272/302, 90%) reported interest‐holder engagement in a single evidence synthesis. The remaining 10% (30/302) are publications providing an account or description of engagement, or methods of engagement, relevant to evidence. We judged 19% (51/272) of evidence syntheses to provide a comprehensive description of engagement, and we have described these, providing examples of how interest‐holders have been engaged in a range of evidence syntheses.

**Objective 2. Describe the methods that have been used to engage interest‐holders in evidence syntheses (see objectives 2a–2h):**

**Objective 2a. Who was engaged?**

Of 272 evidence syntheses in which there was interest‐holder engagement, around three‐quarters involved patients and/or carers, while 17% did not involve patients and/or carers, and for the remainder, this information was unclear. For the “green” evidence syntheses, judged to have the most comprehensive reporting, 84% (43/51) engaged patients/caregivers, although it was most common to engage both patients/caregivers and other interest‐holders (professionals)(30/51, 59%). The professional interest‐holders were most likely to be providers (95% of publications reporting this information, 33/35), but engagement of principal investigators, producers/commissioners, program managers, policy makers, and payers was also reported. Less than half of the “green” evidence syntheses described any demographic characteristics of the interest‐holders, with small numbers presenting some information about gender, age, place or residence, occupation, race/ethnicity/culture, disability, and/or education.

**Objective 2b. How were they invited to be engaged?**

The method of recruitment tended to be very poorly reported. Even in the “green” evidence syntheses, with the most comprehensive reporting, one‐third (17/51) did not clearly report how interest‐holders were invited to be engaged. Most commonly, interest‐holders were recruited using a “closed” group (30/51, 59%), while few (3/51, 6%) reported use of an “open” approach to recruitment.

**Objective 2c. Where were they engaged (geographical and cultural context)?**

Most interest‐holders were engaged from English‐speaking countries with over three‐quarters of all evidence syntheses (215/272, 79%) from the United Kingdom, the United States, Canada, or Australia, and 84.3% (43/51) of the “green” publications from Europe or North America.

**Objective 2d. Why were they engaged (what was the aim of engagement)?**

The stated aim of engagement (if provided) was often specifically related to gaining perspectives of interest‐holders on the topic of the evidence synthesis. Around one‐fifth (60/272) of the evidence syntheses focussed on evidence relating to mental, behavioral, or neurodevelopmental disorders, and 14% (38/272) focussed on neoplasms. Sixteen percent (45/272) focussed on a specific intervention (but not population), and around 12% (32/272) had a focus on research or research methods. However, in many cases, the aim of engagement was expressed in relation to the roles and activities in which interest‐holders were engaged, and to the stage in the evidence synthesis process (see Objectives 2e and 2f below).

**Objective 2e. What did they do (how they were engaged)?**

**Objective 2f. When in the review process were they engaged?**

Information extracted from all identified 272 evidence syntheses was limited to the stage of the review process and the focus of the evidence synthesis. In 42% (113/272), there was a “top and tail” approach, while in around one quarter (67/272) of evidence syntheses, the stage of the review process was unclear. Exploration of the 51 “green” evidence syntheses demonstrated that engagement was reported at all stages and within all review activities, but was most common at the stages of interpretation of findings and data synthesis. As above, this was frequently a “top and tail” approach. Generally, the engaged interest‐holders attended meetings, supplemented with emails/electronic communication, and formal research methods were rarely used.

**Objective 2g. What level of influence, or control, did they have in decision‐making?**

We made judgments on the potential level of influence or control for the 51 “green” evidence syntheses. Most commonly, interest‐holders were judged to have some influence over decisions and outcomes of each activity. For a small number of evidence syntheses, there was engagement in activities for which it was judged that the interest‐holders may have had overall control over final decisions (possibly sharing equal power with the research team). There were no obvious patterns in relation to different forms of engagement, methods used, or level of control during different stages of an evidence synthesis.

**Objective 2h. Were there processes, strategies, or tools to support engagement and evaluation of impact?**

We explored reported processes, strategies, or tools for the 51 “green” evidence syntheses. Twelve percent (6/51) used the ACTIVE framework, and 22% (11/51) the GRIPP2 checklist. A range of other checklists, tools, or frameworks were used by individual evidence syntheses. One‐third (16/51) of evidence syntheses were judged to provide some reflexivity on the process of engagement. In over half of these evidence syntheses (27/51), interest‐holders were co‐authors, but there was substantial variation in the roles and responsibilities of interest‐holder co‐authors.

### 8.2 Comparison to Previous Review Findings

Supporting Information S3 provides results from the previous scoping review and this updated scoping review. The previous review identified 291 evidence syntheses that described interest‐holder engagement, of which 60% were judged to provide few details and/or an inadequate description of the method of engagement. Of the 272 newly identified evidence syntheses, only 29% were judged to provide similarly few details. This suggests that the quality of reporting of interest‐holder engagement may have improved in evidence syntheses published from 2017 onwards. However, while there was some increase in the proportion of evidence syntheses considered to provide a comprehensive account of the methods of engagement (from 11% to 19%), the majority (52%) of the new evidence syntheses in the updated review were judged to fall in the “amber” category. Only 29% of evidence syntheses in the original review clearly engaged patients/carers/family members as interest‐holders, but this increased to 74% amongst the newly identified evidence syntheses, indicating a potential change in practice over time, with the engagement of patients or public becoming more common amongst those evidence syntheses which report interest‐holder engagement.

The original review noted inadequate reporting of key features, even amongst the “green” evidence syntheses, which were considered to provide the most comprehensive reporting. This pattern of inadequate reporting for features such as financial compensation and ethical approval remains common. However, a small number of the new “green” evidence syntheses did report comprehensive information, supported by the ACTIVE Framework and/or GRIPP2 guidance (e.g., [5, 20]). These evidence syntheses suggest that the use of existing tools can enhance the quality and consistency of reporting.

Compared to the previous review, we observed an increase (from 6.8% to 41.4% of included evidence syntheses) in the use of a “top and tail” approach. A possible explanation for this is that the concept of engaging interest‐holders using a “top and tail” approach was first described within the ACTIVE framework [5], which was published after the previous review (building on the results of that review [12]); this may have prompted more researchers to adopt this approach to engagement when conducting evidence syntheses.

### 8.3 Comparison to Evidence Relating to Engagement in Randomized Controlled Trials (RCTs)

The findings of this scoping review align with descriptions of poor reporting of interest‐holder engagement within other types of health research [343]. A recent evaluation of interest‐holder engagement in 360 RCTs (2015–2025) found that, in the 18% that reported on engagement, descriptions of “specific roles and contributions were generally broad and vaguely described” [344]. Of the RCTs reporting engagement, only 15% (10/64) were judged to have “highly detailed” descriptions, with 45% (29/64) having “moderately detailed” and 39% (25/64) “not detailed” descriptions [344]. This broadly aligns with our judgments of 19% “green,” 52% “amber,” and 29% “red,” suggesting similar patterns of poor reporting of descriptions of engagement in RCTs and evidence syntheses. Similar to our findings, Vanneste et al. [344] found that engagement could either occur “throughout” or within specific stages of research planning, conduct, or reporting. However, in contrast to our finding that engagement was most common during the data analysis/synthesis and interpretation of findings stages of an evidence synthesis, within RCTs, engagement was most common during the development phase, suggesting that the study design is influencing decisions about when to engage people. The conclusions from this evaluation of RCTs align with the recommendations supported by our scoping review, focussing on the necessity for clear, detailed reporting and use of standardized reporting tools [344]. Vanneste et al. [344] also reported an inconsistency in the reporting of engagement in the protocol and final report; this is something that we did not explore, and that may merit further investigation in relation to evidence syntheses.

### 8.4 Implications: Approaches to Engagement of Interest‐Holders in Evidence Syntheses

The evidence that we have synthesized demonstrates that there are many different approaches to engagement and that interest‐holders have been involved at all stages of evidence syntheses. Despite the review team hypothesizing potentially different patterns of engagement between types or the focus of evidence synthesis, we found no evidence of such patterns. For example, we considered that realist reviews and scoping reviews may be likely to have unique patterns of engagement as interest‐holder engagement is a recognized methodological component for these types of evidence syntheses. The lack of observable patterns and high level of heterogeneity in approaches to interest‐holder engagement in evidence synthesis could be due to a range of factors, such as the review topic and question (and existing knowledge in that field), the funding/resources available and timelines, and the values, knowledge, and experience of the research team. While we found no generalizable evidence about how different approaches may impact the evidence synthesis, we did find that it is *most common* to:

- Form a “closed” group of interest‐holders (where interest‐holders are invited to take part, and these individuals form the membership of a group which contributes at one or more stage(s) of the review).
- Engage both patients/caregivers and other interest‐holders (professionals).
- Engage interest‐holders at the start and/or end stages of an evidence synthesis.
- Engage interest‐holders in multiple activities throughout the review process (e.g., activities to clarify review question or inclusion criteria, identify outcomes, interpret findings, and/or plan dissemination).
- Engage interest‐holders in “meetings,” without using any formal methods of engagement, and supplement these using email communication.
- Enable interest‐holders to have some influence over decisions and outcomes for specific activities, with the sharing of power reported less commonly.
- Have one or more interest‐holders as a co‐author.

While the above lists what we found to be most common in relation to interest‐holder engagement in evidence synthesis, it is important to highlight the considerable variability between the examples we have explored. The extent of this may be exaggerated by the inconsistency in terminology used to describe the individual approaches to engagement. Reflecting across the different reported approaches, in relation to “what happened,” rather than the terms used within publications to describe this (e.g., a “PPI group,” a “reference group,” etc.), we can observe some distinguishable approaches to who may be engaged, and how they are recruited. These include:

1. Small number, often one or two, of people with lived experience are engaged as “co” researchers, working in partnership with the review team (in a manner which could be described as “co‐production”). These interest‐holders are often co‐authors on publications (and may be co‐grant‐holders on review funding). In many cases, the people engaged are known to the review team and have had previous engagement experience.
2. A group of interest‐holders, often in the range of 6–12 people, but sometimes more (maximum of 28 people within a group within the “green” evidence syntheses [270]), formed specifically as an “advisory” or “reference” group or panel for the evidence synthesis. The recruitment is generally closed, with the review team aiming to form a group that includes interest‐holders with specific knowledge, expertise, or experience. This group could be:
  a. A “patient and public” group, comprising only people with lived experience or the families, carers, or representatives of those with lived experience.
  b. A mixed group, comprising both patients/public and professional interest‐holders (most often “providers” of healthcare).
3. Open engagement of interest‐holders, often for a single workshop or activity (e.g., a survey), in which interest‐holders are engaged but no “group” is formed. This approach to engagement is often conducted in addition to one of the above approaches and is likely to have a specific aim in relation to the evidence synthesis (e.g., to select the outcomes, to explore the interpretation of findings).

**Figure 2:**
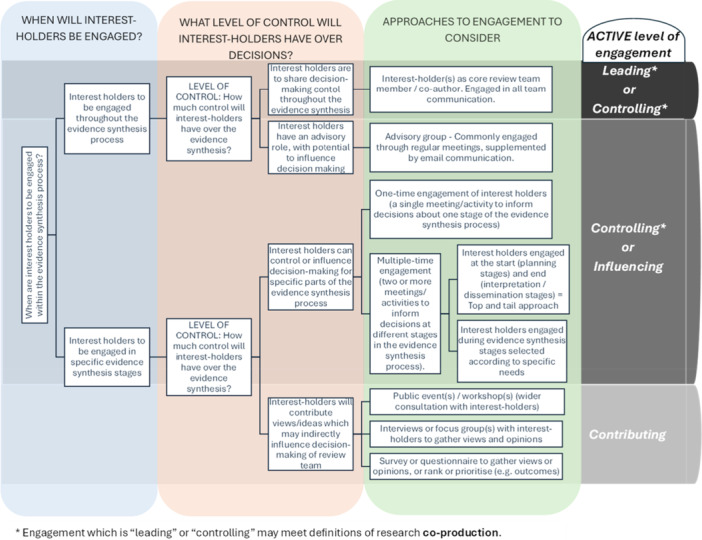
ACTIVE tool to inform the selection of different approaches to engagement. This illustrates potential relationships between when interest‐holders are engaged, the level of control they have over decision making, the approach to engagement which is used, and how this may relate to the level of engagement (using previously defined terms [5]). Taken from left to right, this provides a possible route to inform the selection of different approaches to engagement.

Further, it is clear that during the planning of interest‐holder engagement, a number of decisions are made by the teams responsible for the planning and/or conduct of the evidence synthesis. These decisions include consideration of several factors, including (but not limited to) the stages of the evidence synthesis at which interest‐holders will be engaged, the roles the interest‐holders will have, and, implicit to this, the level of control that interest‐holders may hold over decision‐making in relation to specific parts of the evidence synthesis process. The decisions that are made in relation to these domains may be linked to the engagement approach that is selected. Conversely, within some published evidence syntheses, it appears possible that the approach to engagement was selected first, and this then dictated the level of control and stages of the evidence synthesis at which interest‐holders were involved. The potential relationship between when interest‐holders are engaged, the level of control that they have over decisions relevant to the evidence synthesis, and the approach to engagement is illustrated in Figure 2 (“ACTIVE Tool” to select engagement approach). This figure provides a potential tool to support the selection of approaches to engagement within an evidence synthesis. The examples identified in this scoping review demonstrate that it is common to engage interest‐holders both “throughout” the evidence synthesis process (i.e., the “top” horizontal route in Figure 2) and also within specific evidence synthesis activities (i.e., the “bottom” horizontal route in Figure 2). Thus, there may be more than one “approach” to engagement selected for a single evidence synthesis.

### 8.5 Strengths and Limitations

#### 8.5.1 Scoping Review Objectives

Our scoping review provides an overview of the current state of interest‐holder engagement in evidence syntheses and gives descriptions of different examples of interest‐holder engagement in evidence syntheses. However, this scoping review did not explore the impact or usability of different engagement methods and, consequently, is not able to provide specific recommendations relating to “best” ways of engaging interest‐holders in evidence syntheses. We only included publications in which interest‐holders had an active role during the planning, conduct, or reporting of an evidence synthesis; we did not include publications reporting on strategies to inform interest‐holders about the evidence synthesis, in which interest‐holders received information only. Sharing of information by review teams with interest‐holders could arguably be considered a useful form of engagement, but this was beyond the scope of our work. Our objective focussed on healthcare evidence syntheses, but our pre‐stated eligibility criteria explicitly included evidence syntheses relating to social care topics; this scoping would have been strengthened by greater clarity in this regard.

#### 8.5.2 Identification of Relevant Publications and Those With the Most Comprehensive Description

Although we implemented a robust and comprehensive search of electronic databases and used two independent reviewers at all stages of study selection, a key limitation of this scoping review is that we are unlikely to have comprehensively identified all relevant publications. This is because poor (and absent) reporting and inconsistent terminology mean we will not have identified some evidence syntheses in which interest‐holders were engaged. While this is a limitation of our scoping review data, we believe that our strategy of selecting the evidence syntheses with the most “comprehensive” description of interest‐holder engagement is a strength. This subset of “green” evidence syntheses is not designed to be comprehensive, nor to reflect quality of engagement, but rather to provide examples from which lessons can potentially be learned in relation to methods of engaging interest‐holders within evidence syntheses.

#### 8.5.3 Poor Reporting of Key Aspects of Engagement

A strength of this updated scoping review was that we were able to build on the findings of and learning from the previous version of this scoping review. Thus, we extracted data according to domains identified by the ACTIVE Framework [5], as well as those considered important by a wide range of interest‐holders within the MuSE consortium who contributed to the development of the updated protocol. However, despite the clearly defined domains of interest for our scoping review, findings and conclusions were limited by poor reporting within the included publications. Importantly, we did find evidence of improvements in reporting between the publications identified by the previous and updated reviews, for example, in reporting details of the interest‐holders (previous version was unclear for 30%; updated version unclear for 10%), stage of evidence synthesis (previous version was unclear for 48%; updated version unclear for 25%). This improvement in reporting is also reflected in the proportion of publications judged to provide few or inadequate details (“red”), which decreased from 60% in the previous version of this review to 29% for this updated version. While these findings suggest an improvement in reporting, it is not possible to determine if there have been any changes in the absence of reporting (i.e., evidence syntheses in which there was engagement, but for which there is no information reported).

However, despite the observed improvements in reporting, even amongst the publications judged to provide the most comprehensive description (i.e., “green” publications) there remain some domains for which reporting seriously limits conclusions being drawn; for example, of the “green” publications, less than half provided any information about ethics or ethical approval (56% not reported), payment of expenses (78% not reported), other forms of compensation (75% not reported), equity‐related characteristics of interest‐holders (51% reported no information on any domains of PROGRESS‐Plus). One‐third of the “green” publications reported that there was interest‐holder engagement “throughout” the evidence synthesis, but did not provide any further details; this limits our understanding of the activities that interest‐holders were engaged in. Similarly, while over half of the publications list an interest‐holder as a co‐author, many do not detail the specific roles of the interest‐holder co‐authors. New mandatory requirements from many funders and journals for reporting of patient and public engagement could be an important factor in changes in reporting of engagement over time.

Exploration of the journals that published the “green” publications could potentially provide some insight into factors that influence comprehensiveness of reporting (see Table 9). However, without systematic consideration of issues such as the number of evidence syntheses published and journal word count, any interpretation must be cautious. Almost two‐fifths (9/51, 18%) of “green” papers were published in the journal *Health Expectations*; this journal focusses on publishing articles directly relating to patient and public involvement and engagement, and reporting of engagement in published studies is mandatory, providing a logical explanation for the large proportion meeting our criteria for providing comprehensive descriptions of engagement. Only slightly fewer (8/51, 16%) were published in the NIHR Journals Library, where reports often have large word counts and where there is a mandatory requirement for all publications to “explain how patients and the public have been involved in the study outside of being study participants” [345], with recommendations to use the GRIPP2 reporting tool [20]. The next two journals publishing the greatest numbers of “green” evidence syntheses (three each) both explicitly support engagement, although reporting is not mandatory. The remaining 26 journals all published one or two “green” evidence syntheses; we were unable to identify any information relating to reporting of interest‐holder engagement within author guidelines for any of these journals. This evidence supports the value of journals mandating reporting of interest‐holder engagement and/or having explicit statements or policies supporting the concept of interest‐holder engagement. Further systematic exploration of author guidelines, journal reporting requirements, and comprehensiveness of the reporting may be beneficial in informing approaches to improve reporting in future publications.

A key limitation of our work is that we were dependent on the reporting provided in publications, many of which had a primary aim of reporting the findings of a specific evidence synthesis. Not only does this mean that our conclusions were limited by the lack of information provided by authors on interest‐holder engagement, but it also means that we are unable to make any assessment of whether the reported engagement is an accurate representation of what happened. Further, under‐reporting of engagement will mean that we will not have identified all evidence syntheses in which there was engagement. We also recognize that there is a risk that some articles may have overstated the amount and influence of patient and public involvement, and that the interest‐holder engagement may have been largely tokenistic in evidence syntheses where reporting is poor.

#### 8.5.4 Equity‐Related Characteristics

We explored whether information on equity‐related characteristics of the engaged interest‐holders was reported. However, we did not explicitly gather data on whether review teams specifically sought to engage interest‐holders with diverse characteristics, experiences, or opinions.

#### 8.5.5 Geographical Location of Interest‐Holders Engaged

A strength of our findings is that we identified publications reporting interest‐holder engagement from at least 30 different countries. However, a large majority are from English‐speaking and/or high‐income countries, potentially limiting the applicability of findings to other contexts and affecting the generalizability of findings.

## 9 Conclusions

Our international team from the MuSE consortium has updated a previous scoping review, bringing together up‐to‐date evidence relating to interest‐holder engagement in evidence syntheses. We provide an overview of 302 publications and describe in more detail the methods used by 51 publications, which we judged to provide the most comprehensive description of methods of interest‐holder engagement. Many approaches to engagement have been used, with no evidence of patterns according to different types or focus of evidence syntheses. Interest‐holders have been engaged at all stages of the evidence synthesis process. We conclude that there may be a potential relationship between when interest‐holders are engaged, the level of control they have over decision‐making, and the approach to engagement that is used. This information may usefully inform the selection of different approaches to engagement in evidence synthesis. Conclusions arising from this scoping review are limited by poor reporting of interest‐holder engagement in evidence syntheses, and improvements in transparent reporting are essential. Most evidence syntheses engaging interest‐holders originate from a small number of high‐income countries, potentially limiting the generalizability of the findings.

Our team considers the following recommendations to be supported by the findings of this scoping review. Those *conducting* evidence syntheses should adhere to relevant standards and principles related to good practice in engagement in research (e.g., UK Standards for Public Involvement [13]), involve key interest‐holders as early as possible, and pre‐plan their engagement approach, with clear terms of reference relating to roles, responsibilities, and issues such as compensation and acknowledgment. Additionally, they should consider the topic, resources, and timeline when planning engagement, as well as the types and characteristics of interest‐holder and their relationship to underrepresented or marginalized groups. This aligns with a growing awareness of the importance of considering these personal characteristics, both when engaging interest‐holders in research [346] and when conducting evidence syntheses [347, 348]. The ACTIVE Framework may be used to guide decisions on who to involve and at what stages, and our newly proposed ACTIVE Tool (Figure 2) may support the selection of an appropriate engagement approach, but testing of this is required. When *reporting* an evidence synthesis, it is essential that key elements of interest‐holder engagement are transparently documented. The ACTIVE Framework [5] and GRIPP2 Guidance [20] provide useful resources to support reporting. We encourage journals to proactively mandate reporting of interest‐holder engagement, as seen in Cochrane's recent requirement for reporting consumer engagement in systematic reviews [349]. We recommend that reporting guidelines, such as PRISMA [350] and PRISMA extensions [351], be updated to include the reporting of interest‐holder engagement, covering who was engaged, at what stages, and what activities or decisions they contributed to. It is important that the roles of all co‐authors, including interest‐holders, should be reported following the CREDIT contributor taxonomy [352], and that the conflicts of interest (if any) of all interest‐holders are disclosed. Recommendations for future research include the development of tools to support the planning and conduct of interest‐holder engagement in evidence syntheses and research on the impact and usability of different engagement approaches. Future research should also assess the effects on engaged individuals, research teams, project resources, timelines, and the overall evidence synthesis and dissemination process. It is important that funding bodies are cognizant of and that research teams have access to adequate resources to support meaningful interest‐holder engagement in evidence syntheses. These recommendations are summarized in Box 3.

Box 3 **Recommendations supported by the findings of this scoping review.**

- Recommendations for engagement of interest‐holders in evidence syntheses. Those conducting evidence syntheses should:
  ∘ Adhere to relevant standards and principles relating to engagement in research (e.g., [13, 338]).
  ∘ Engage key interest‐holders (even if this is just 1 or 2 people) at the earliest opportunity.
  ∘ Pre‐plan their engagement approach.
  ∘ Consider the evidence synthesis topic, resources, and timeline when planning engagement.
  ∘ Consider using the ACTIVE Framework [5] as a tool to support decisions relating to who to involve, at what stages in the review, and in what way, and the ACTIVE Tool (Figure 2) to support selection of an approach to engagement of interest‐holders within a specific evidence synthesis.
  ∘ Consider factors relating to diversity, equity, and inclusion when planning and engaging interest‐holders.
  ∘ Have access to adequate resources to support interest‐holder engagement (funding bodies should provide appropriate resources to enable meaningful engagement).
- Recommendations relating to the reporting of engagement of interest‐holders in evidence syntheses.
  ∘ It is essential that evidence synthesis authors report key elements of interest‐holder engagement within final reports. The ACTIVE Framework [5] and GRIPP2 Guidance [20] may be useful in guiding this. Journals should be proactive in mandating this reporting. Cochrane has recently introduced new mandatory reporting of consumer involvement within Cochrane systematic reviews [349].
  ∘ Reporting guidelines aimed at enhancing the quality and transparency of healthcare evidence syntheses, including PRISMA [350] and PRISMA extensions [351], should be updated to include the reporting of interest‐holder engagement.
  ∘ Conflicts of interest (if any) of interest‐holders should be reported.
  ∘ Roles of all co‐authors, including those who are interest‐holders, should be reported according to the CREDIT contributor taxonomy [352].
- Recommendations for research. There is a need to:
  ∘ Develop tools to further support the planning and conduct of interest‐holder engagement in evidence syntheses; these should focus on practical elements of engagement.
  ∘ Conduct research to explore the impact and usability of different engagement approaches, considering the impact on the individuals engaged, as well as the research team, project resources, and timelines, and the final evidence synthesis and dissemination activities.
  ∘ Consider how project resources impact decisions around interest‐holder engagement, and how to prioritize engagement activities when resources are limited.

The MuSE Consortium is currently developing guidance for interest‐holder engagement [7], which will build on the findings and recommendations of this review; the planned guidance has the potential to provide clear evidence‐informed recommendations to support both conduct and reporting in this field.

## Author Contributions

**Alex Todhunter‐Brown:** conceptualization, funding acquisition, methodology, data curation, investigation, formal analysis, visualization, writing – review and editing, writing – original draft, project administration, supervision. **Jennifer Petkovic:** conceptualization, funding acquisition, methodology, data curation, investigation, writing – original draft, writing – review and editing, project administration, supervision. **Christine Chang:** methodology, investigation, formal analysis, writing – original draft, writing – review and editing. **Ursula Griebler:** investigation, formal analysis, writing – original draft, writing – review and editing. **Ailish Hannigan:** investigation, formal analysis, writing – original draft, writing – review and editing, methodology. **Jennifer Hilgart:** methodology, investigation, formal analysis, writing – original draft, writing – review and editing. **Basharat Hussain:** investigation, formal analysis, writing – original draft, writing – review and editing. **Janet Jull:** investigation, formal analysis, methodology, writing – original draft, writing – review and editing. **Christina Koscher‐Kien:** methodology, investigation, formal analysis, writing – original draft, writing – review and editing, data curation. **Dominic Ledinger:** data curation, investigation, formal analysis, writing – original draft, writing – review and editing. **Barbara Nussbaumer‐Streit:** investigation, formal analysis, writing – original draft, writing – review and editing. **Oyekola Oloyede:** methodology, data curation, investigation, formal analysis, writing – original draft, writing – review and editing. **Eve Tomlinson:** methodology, investigation, formal analysis, writing – original draft, writing – review and editing. **Shoba Dawson:** methodology, investigation, writing – review and editing. **Omar Dewidar:** methodology, investigation, writing – review and editing. **Sean Grant:** investigation, writing – review and editing. **Lyubov Lytvyn:** investigation, writing – review and editing. **Thomas W. Concannon:** methodology, writing – review and editing. **Leonila Dans:** methodology, writing – review and editing. **Denny John:** methodology, writing – review and editing. **Zoe Jordan:** methodology, writing – review and editing. **Evan Mayo‐Wilson:** methodology, writing – review and editing. **Chris McCutcheon:** methodology, writing – review and editing. **Francesco Nonino:** methodology, writing – review and editing. **Danielle Pollock:** methodology, investigation, writing – review and editing. **Karine Toupin April:** methodology, writing – review and editing. **Pauline Campbell:** conceptualization, funding acquisition, methodology, data curation, writing – review and editing, project administration, supervision. **Joanne Khabsa:** methodology, writing – review and editing, project administration, supervision. **Olivia Magwood:** methodology, data curation, investigation, writing – review and editing, project administration, supervision. **Vivian Welch:** funding acquisition, conceptualization, methodology, writing – review and editing, supervision, project administration. **Peter Tugwell:** conceptualization, methodology, funding acquisition, writing – review and editing, project administration, supervision.

## Ethics Statement

The protocol for this scoping review is available at https://osf.io/8t6jn (published 23/02/2023). This scoping review is reported according to PRISMA‐ScR Checklist (available on request) and GRIPP2 template (provided as Appendix 2).

## Conflicts of Interest

Alex Todhunter‐Brown: Co‐lead of Cochrane Coproduction Methods Group (unpaid role). Christine Chang: Participation in US Food and Drug Administration, Drug Safety Oversight Board (unpaid role); Member of the Board of Trustees of Guidelines International Network (payment for travel to meetings received). Janet Jull: Canada Research Chair (some funding for Canadian Institute of Health Research); grant funding received from Canadian Institutes of Health Research, Social Sciences and Humanities Research, and New Frontiers Research (all Canadian Tri‐Council funders). Dominic Ledinger: PhD scholarship from Gesellschaft für Forschungsföderung Niederösterreich m.b.H., project number FTI23‐D‐042; PhD topic: Co‐production and knowledge user involvement in evidence synthesis. Sean Grant: Spouse is an employee of Eli Lilly and owns stock. Thomas W. Concannon ‐ recipient of grants or contracts from: Centers for Disease Control and Prevention/NIOSH and National Institutes of Health/NCAT. Zoe Jordan: Royalties received from Wolters Kluwer Health for the sale of JBI tools and resources to support evidence based decision making. Evan Mayo‐Wilson: Grant funding received from Agency for Healthcare Research and Quality (AHRQ), National Institutes of Health (NIH), Knowledge Works Global (subcontract with Patient Centered Outcomes Research Institute (PCORI)). Payment/Honoraria from National Institutes of Health; support for travel/attending meetings from Monash University. Danielle Pollock: Grant funding from Stillbirth CRE /Stillbirth Foundation. Peter Tugwell: Consulting fees from Reformulary Group (Providing independent medical consultation professional services to the firms listed in this section); I am [unpaid] Chair of the Management Group of a registered non‐profit independent medical research organization, OMERACT, whose goal is to improve and advance the health outcomes for patients suffering from musculo skeletal conditions. OMERACT receives arms‐length funding from 11 companies (Abbvie, Astra Zenaca, Aurinia, BMS, Centrexion, GSK, Horizon PharmaInc, Janssen, Novartis, Pfizer & Sparrow). Other authors declare no conflicts of interest.

## Supporting information

Supplementary Material 1, Table of included studies (*n* = 302).

Supplementary Material 2, How were interest‐holders engaged (*n*=51).

Supplementary Material 3, Comparison with previous scoping review results.

PRISMA‐ScR‐Fillable‐Checklist.

## Data Availability

Data sharing is not applicable to this article as no datasets were generated or analyzed during the current study.

## Appendix 1 Differences Between Protocol and Review

**Searching**

- Protocol stated plans for gray literature searching and use of citation chaser tool. These were not conducted due to the volume of evidence identified through database searches.

**Data extraction and coding**

New domains added to data extraction form:

- Type of publication
- Terminology used to describe methods of engagement
- Form of governance
- Reflexivity
- Conflicts of interest

New/amended codes added to the following pre‐planned domains:

- Type of evidence synthesized
- Form of engagement
- Methods used to facilitate engagement
- Level of involvement (at each stage)
- Compensation
- Tool or method of reporting involvement

For further details of differences between protocol and review for data extraction and coding, see Appendix 6.

**Synthesis/results**

We planned to report details of the interest‐holders included in the new “Green” evidence syntheses according to the 11 different categories of interest‐holders. However, the lack of clear reporting of interest‐holder categories led us to deviate from this and instead group interest‐holders into broader categories.

## Appendix 2 Engagement of interest‐holders in the scoping review: Description using GRIPP2 reporting template [20]

**Table:**

GRIPP2 (short form) topic/Item	Response
1: Aim/Report the aim of PPI in the study	This review was conducted as one of a series of evidence syntheses focussed on interest‐holder engagement in evidence synthesis produced by the MuSE Consortium. The MuSE Consortium comprises individuals with an interest in the engagement of people in research and guidelines from a wide variety of backgrounds [353]. Our aim was to ensure that there was input from a wide range of interest‐holders into this review, ensuring that the perspectives of all interest‐holder groups were appropriately reflected within all stages of the review.
2: Methods/Provide a clear description of the methods used for PPI in the study	MuSE Consortium members were given opportunities to volunteer to contribute to this review. Those who volunteered were able to select the role they had in the review, ranging from commenting on drafts of the protocol and paper, to actively contributing to review tasks, including study selection, data extraction, and data synthesis. Appendix 3 lists the categories of interest‐holders which interest‐holders who contributed as authors consider themselves part of. Our author team included interest‐holders who were patients, payers, peer‐review journal editors, principal investigators, producers, and commissioners and providers. None of the authors described themselves as payers, policymakers, or public.
3: Study results/Outcomes—Report the results of PPI in the study, including both positive and negative outcomes	Contributions of interest‐holders meeting ICMJE criteria for authorship [36] are described using the CRediT author statement [35] and detailed under Author Contributions, and those of interest‐holders not meeting authorship criteria are listed in Acknowledgments. The large number of interest‐holder authors, representing a range of interest‐holder experiences, perspectives, and expertise, was a positive outcome. A negative outcome was the lack of interest‐holders who considered themselves payers or policymakers.
4: Discussion and conclusions/Outcomes—Comment on the extent to which PPI influenced the study overall. Describe positive and negative effects	Interest‐holders from the MuSE Consortium were actively engaged in this scoping review. Twenty‐four of the authors were volunteers who responded to initial requests to support the scoping review update. The author team comprises a wide range of different interest‐holders (see Appendix 2) and works within 12 different countries, from 5 continents. A positive effect was the varied perspectives that this large group of interest‐holders brought to the review. Our approach benefitted from team members working in pairs to complete the extraction of the “green” evidence syntheses and to write narrative summaries relating to sub‐groups of these evidence syntheses; however, this meant that there was no single person who contributed across all included evidence syntheses. This may have introduced some inconsistencies across extraction for sub‐groups of the evidence syntheses.
5: Reflections/critical perspective/Comment critically on the study, reflecting on the things that went well and those that did not, so others can learn from this experience	We successfully completed this complex scoping review, working as a large team of volunteer reviewers, from a broad range of backgrounds and different interest‐holder groups. Our regular team meetings were a strength as these provided opportunities for the sharing of ideas in relation to the interpretation of the findings. The large range of interest‐holders contributing to the drafting of the manuscript led to multiple comments, which were time‐consuming to incorporate into the final output, but which we believe substantially enhance the final version of this review. Our approach may have led to some differences between the previous version of this scoping review and this updated version. Unlike the previous version of this scoping review, which was limited by the small number of reviewers involved, this update has been conducted by a large team, with a wide range of different backgrounds and experiences, bringing considerable strength to this version. It is possible that some of the differences observed between data in the previous and updated review may be explained by different interpretations of domains during extraction and coding. For example, the updated review has a greater proportion of “green” evidence syntheses reporting engagement in the analysis and synthesis of data, with 60.8% (31/51) of evidence syntheses, compared to 16.7% (5/30) in the original review. Potentially, this could be due to differences in the categorization of data within the two versions of the review, rather than reflecting a difference in patterns of engagement over time. To mitigate against differing interpretations between pairs of reviewers, we held weekly team meetings to discuss disagreements, challenges, or issues arising during the data extraction and categorization, but the large number of evidence syntheses and volume of data extracted may mean that some variations could still have occurred between pairs of reviewers.

## Appendix 3 Review author identification as interest‐holders

**Table:**

Categories of interest‐holders	Description	Review authors:
Patients, patient caregivers, patient advocates/organizations	Those with lived experience of the condition of interest or who care for or advocate on behalf of those with lived experience	Danielle Pollock, Karine Toupin April, Lyubov Lytvyn, Pauline Campbell
Payers of health research	Individuals and organizations that fund research projects, such as government funders, industry funders, foundations	Christine Chang
Payers/Purchasers of health services	Individuals, organizations, and entities that pay for health services	
Peer‐reviewed journals editors	Those who set journal policy on guidelines and manage the peer review process and editing	Vivian Welch, Peter Tugwell, Sean Grant, Evan Mayo‐Wilson, Jennifer Hilgart, Zoe Jordan
Policymakers	Individuals, organizations, and entities that craft public or private policy (on health) at any level of government	Thomas W. Concannon
Principal investigators and all members of the research team	Individuals, organizations, and associations that conduct or advocate for health research	Alex Todhunter‐Brown, Eve Tomlinson, Francesco Nonino, Zoe Jordan, Janet Jull, Danielle Pollock, Ailish Hannigan, Vivian Welch, Omar Dewidar, Leonila Dans, Karine Toupin April, Chris McCutcheon, Adrienne Stevens, Peter Tugwell, Sean Grant, Oyekola Oloyede, Barbara Nussbaumer‐Streit, Basharat Hussain, Jennifer Petkovic, Evan Mayo‐Wilson, Chirs McCutcheon, Christina Koscher‐Kien, Karine Toupin April, Ursula Griebler, Janet Jull, Dominic Ledinger, Shoba Dawson, Lyubov Lytvyn, Denny John, Zoe Jordan, Joanne Khabsa, Olivia Magwood, Pauline Campbell
Producers and commissioners of guidelines	Institutions and organizations that commission, develop, or implement guideline development procedures	Leonila Dans, Adrienne Stevens
Product makers	Individuals working for companies that manufacture pharmaceuticals, medical devices, medical procedures, health technologies, or for‐profit educational and behavioral packages	
Program managers	Managers/directors who plan, lead, oversee, or deliver any program that provides public health, community services, or clinical care (e.g., budgeting, hiring, staffing, organizing, coordinating, reporting)	
Providers	Persons and their professional associations who provide health care in a professional capacity and are allowed by regulatory bodies to provide a health care service	Francesco Nonino, Oyekola Oloyede, Leonila Dans, Peter Tugwell, Denny John
Public	Individuals in the general population of a defined geographic area, excluding patients, caregivers, and health professionals living or working with the condition of interest	

## Appendix 4 Search strategies for all databases

Ovid MEDLINE(R)

1. Stakeholder Participation/1988
2. patient participation/28561
3. consumer participation/18172
4. Community‐Based Participatory Research/5309
5. Community‐based participatory research.ti,ab,kf.3636
6. (coproduction or co‐production).ti,ab,kf.2836
7. ((stakeholder* or advisor* or reference* or expert* or consultation* or steering) adj2 (group* or panel*)).ti,ab,kf.48206
8. ((stakeholder* or consultant* or patient* or consumer* or public or caregiver* or care‐giver* or communit* or citizen* or user* or service‐user* or end‐user* or clinician* or doctor* or physician* or nurse* or policymaker* or policy‐maker* or funder* or indust* or pharmac* or payer* or provider* or pharmacist*) adj2 (engag* or involv* or input or participat* or collaborat* or cooperat*)).ti,ab,kf.173373
9. or/1‐8258670
10. meta‐analysis.pt.160566
11. meta‐analysis/or systematic review/or meta‐analysis as topic/or “meta analysis (topic)”/or “systematic review (topic)”/or exp technology assessment, biomedical/302807
12. ((systematic$ adj3 (review$ or overview$)) or (methodologic$ adj3 (review$ or overview$))).ti,ab.267102
13. ((quantitative adj3 (review$ or overview$ or synthes$)) or (research adj3 (integrati$ or overview$))).ti,ab.13669
14. ((integrative adj3 (review$ or overview$)) or (collaborative adj3 (review$ or overview$)) or (pool$ adj3 analy$)).ti,ab.34275
15. (data synthes$ or data extraction$ or data abstraction$).ti,ab.35050
16. (handsearch$ or hand search$).ti,ab.10461
17. (mantel haenszel or peto or der simonian or dersimonian or fixed effect$ or latin square$).ti,ab.31850
18. (met analy$ or metanaly$ or technology assessment$ or HTA or HTAs or technology overview$ or technology appraisal$).ti,ab.10285
19. (meta regression$ or metaregression$).ti,ab.12347
20. (meta‐analy$ or metaanaly$ or systematic review$ or biomedical technology assessment$ or bio‐medical technology assessment$).mp,hw.405695
21. (medline or cochrane or pubmed or medlars or embase or cinahl).ti,ab,hw.294627
22. (cochrane or (health adj2 technology assessment) or evidence report).jw.20745
23. (meta‐analysis or systematic review).mp.379456
24. (comparative adj3 (efficacy or effectiveness)).ti,ab.15291
25. (outcomes research or relative effectiveness).ti,ab.8386
26. ((indirect or indirect treatment or mixed‐treatment) adj comparison$).ti,ab.2521
27. (meta‐ethnograph$ or metaethnograph$ or meta ethnograph or meta‐study or metastudy or meta study).ti,ab.858
28. ((qualitative adj3 (review$ or overview$ or synthes$)) or (research adj3 (integrati$ or overview$))).ti,ab.17202
29. (evidence adj2 map$).ti,ab.1217
30. ((evidence or realist) adj3 (review$ or overview$ or synthes$)).ti,ab.76489
31. or/10‐30642113
32. 9 and 3121586

CINAHL

S32, S8 AND S31 (11,454)

S31, S9 OR S10 OR S11 OR S12 OR S13 OR S14 OR S15 OR S16 OR S17 OR S18 OR S19 OR S20 OR S21 OR S22 OR S23 OR S24 OR S25 OR S26 OR S27 OR S28 OR S29 OR S30 (301,653)

S30, TI (((evidence or realist) N2 (review* or overview* or synthes*))) OR AB (((evidence or realist) N2 (review* or overview* or synthes*))) (29,224)

S29, TI evidence N1 map* OR AB evidence N1 map* (437)

S28, TI (((qualitative N2 (review* or overview* or synthes*)) or (research N2 (integrati* or overview*)))) OR AB (((qualitative N2 (review* or overview* or synthes*)) or (research N2 (integrati* or overview*)))) (10,008)

S27, TI ((meta‐ethnograph* or metaethnograph* or meta ethnograph or meta‐study or metastudy or meta study)) OR AB ((meta‐ethnograph* or metaethnograph* or meta ethnograph or meta‐study or metastudy or meta study)) (23,996)

S26, TI (((indirect or indirect‐treatment or mixed‐treatment) W0 comparison*)) OR AB (((indirect or indirect‐treatment or mixed‐treatment) W0 comparison*)) (1,178)

S25, TI (“outcomes research” or “relative effectiveness”) OR AB (“outcomes research” or “relative effectiveness”) (3,942)

S24, TI (comparative N2 (efficacy or effectiveness)) OR AB (comparative N2 (efficacy or effectiveness)) (6,320)

S23, TI (meta‐analys#s or (systematic w0 review*)) OR AB (meta‐analys#s or (systematic w0 review*)) OR MW (meta‐analys#s or (systematic w0 review*)) (213,728)

S22, SO cochrane or (health* N1 (technology W0 assessment*)) or (evidence W0 report*) (9,531)

S21, TI ((medline or cochrane or pubmed or medlars or embase or cinahl)) OR AB ((medline or cochrane or pubmed or medlars or embase or cinahl)) OR MW ((medline or cochrane or pubmed or medlars or embase or cinahl)) (160,626)

S20, TI (meta‐analy* or metaanaly* or (systematic W0 review*) or “biomedical technology assessment” or “biomedical technology assessments” or “bio‐medical technology assessment” or “bio‐medical technology assessments”) OR AB (meta‐analy* or metaanaly* or (systematic W0 review*) or “biomedical technology assessment” or “biomedical technology assessments” or “bio‐medical technology assessment” or “bio‐medical technology assessments”) OR MW (meta‐analy* or metaanaly* or (systematic W0 review*) or “biomedical technology assessment” or “biomedical technology assessments” or “bio‐medical technology assessment” or “bio‐medical technology assessments”) (214,962)

S19, TI ((meta W0 regression*) or metaregression*) OR AB ((meta W0 regression*) or metaregression*) (4,616)

S18, TI ((met W0 analy*) or metanaly* or (technology W0 assessment*) or HTA or HTAs or (technology W0 overview*) or (technology W0 appraisal*)) OR AB ((met W0 analy*) or metanaly* or (technology W0 assessment*) or HTA or HTAs or (technology W0 overview*) or (technology W0 appraisal*)) (4,623)

S17, TI ((mantel W0 haenszel) or peto or (der W0 simonian) or dersimonian or (fixed W0 effect*) or (latin W0 square*)) OR AB ((mantel W0 haenszel) or peto or (der W0 simonian) or dersimonian or (fixed W0 effect*) or (latin W0 square*)) (9,483)

S16, TI (handsearch* or hand‐search*) OR AB (handsearch* or hand‐search*) (4,892)

S15, TI ((data W0 synthes*) or (data w0 extraction*) or (data W0 abstraction*)) OR AB ((data W0 synthes*) or (data w0 extraction*) or (data W0 abstraction*)) (13,846)

S14, TI ((integrative N2 (review* or overview*) or (collaborative N2 (review* or overview*)) or (pool* N2 analy*)) OR AB ((integrative N2 (review* or overview*) or (collaborative N2 (review* or overview*)) or (pool* N2 analy*)) (16,854)

S13, TI ((quantitative N2 (review* or overview* or synthes*) or (research N2 (integrati* or overview*))) OR AB ((quantitative N2 (review* or overview* or synthes*) or (research N2 (integrati* or overview*))) (6,175)

S12, TI ((systematic* N2 (review* or overview*) or (methodologic* N2 (review* or overview*))) OR AB ((systematic* N2 (review* or overview*) or (methodologic* N2 (review* or overview*))) (137,240)

S11, MH Systematic Review (112,124)

S10, MH Meta Analysis (64,175)

S9, PT meta‐analysis (4,226)

S8, S1 OR S2 OR S3 OR S4 OR S5 OR S6 OR S7 (116,320)

S7, TI ((stakeholder* or consult* or patient* or consumer* or client* or public or caregiver* or care‐giver* or communit* or citizen* or user* or service‐user* or end‐user* or clinician* or doctor* or physician* or nurs* or policymaker* or policy‐maker* or funder* or indust* or pharmac* or payer* or provider* or carer or carers or healthcare* or health‐carer* or healthprovider* or health‐provider* or frontline* or (lab* N0 technician*) or facult* or frontliner# or front‐liner#) N1 (engag* or involv* or input# or collaborat* or cooperat*)) OR AB ((stakeholder* or consult* or patient* or consumer* or client* or public or caregiver* or care‐giver* or communit* or citizen* or user* or service‐user* or end‐user* or clinician* or doctor* or physician* or nurs* or policymaker* or policy‐maker* or funder* or indust* or pharmac* or payer* or provider* or carer or carers or healthcare* or health‐carer* or healthprovider* or health‐provider* or frontline* or (lab* N0 technician*) or facult* or frontliner# or front‐liner#) N1 (engag* or involv* or input# or collaborat* or cooperat*)) OR MW ((stakeholder* or consult* or patient* or consumer* or client* or public or caregiver* or care‐giver* or communit* or citizen* or user* or service‐user* or end‐user* or clinician* or doctor* or physician* or nurs* or policymaker* or policy‐maker* or funder* or indust* or pharmac* or payer* or provider* or carer or carers or healthcare* or health‐carer* or healthprovider* or health‐provider* or frontline* or (lab* N0 technician*) or facult* or frontliner# or front‐liner#) N1 (engag* or involv* or input# or collaborat* or cooperat*)) (69,794)

S6, TI ((stakeholder* or advisor* or reference* or expert* or steering) N1 (group* or panel* or organi#ation* or body or lobb* or part*)) OR AB ((stakeholder* or advisor* or reference* or expert* or steering) N1 (group* or panel* or organi#ation* or body or lobb* or part*)) OR MW ((stakeholder* or advisor* or reference* or expert* or steering) N1 (group* or panel* or organi#ation* or body or lobb* or part*)) 26,428)

S5, TI (coproduction or co‐production) OR AB (coproduction or co‐production) OR MW (coproduction or co‐production) (790)

S4, TI Community‐based participatory research OR AB Community‐based participatory research OR MW Community‐based participatory research (2,029)

S3, MH Professional Practice, Research‐Based OR MH Occupational Therapy Practice, Research‐Based OR MH Physical Therapy Practice, Research‐Based (2,255)

S2, MH Consumer Participation (23,024)

S1, MH Stakeholder Participation (2,020)

SCOPUS

(TITLE‐ABS (“Community‐based participatory research” OR (coproduction OR co‐production) OR ((stakeholder* OR advisor* OR reference* OR expert* OR consult* OR steering) W/1 (group* OR panel* OR organi*ation* OR body OR lobb* OR part*)) OR ((stakeholder* OR consult* OR patient* OR consumer* OR client* OR public OR caregiver* OR care‐giver* OR communit* OR citizen* OR user* OR service‐user* OR end‐user* OR clinician* OR doctor* OR physician* OR nurs* OR policymaker* OR policy‐maker* OR funder* OR indust* OR pharmac* OR payer* OR provider* OR carer OR carers OR healthcare* OR health‐carer* OR healthprovider* OR health‐provider* OR frontline* OR frontliner* OR front‐liner*) W/1 (engag* OR involv* OR input* OR collaborat* OR cooperat* OR advice OR advise OR feeback))) OR INDEXTERMS (“Community‐based participatory research” OR (coproduction OR co‐production) OR ((stakeholder* OR advisor* OR reference* OR expert* OR consult* OR steering) W/1 (group* OR panel* OR organi*ation* OR body OR lobb*)) OR ((stakeholder* OR consult* OR patient* OR consumer* OR client* OR public OR caregiver* OR care‐giver* OR communit* OR citizen* OR user* OR service‐user* OR end‐user* OR clinician* OR doctor* OR physician* OR nurs* OR policymaker* OR policy‐maker* OR funder* OR indust* OR pharmac* OR payer* OR provider* OR carer OR carers OR healthcare* OR health‐carer* OR healthprovider* OR health‐provider* OR frontline* OR frontliner* OR front‐liner*) W/1 (engag* OR involv* OR input* OR collaborat* OR cooperat*)))) AND ((TITLE‐ABS (((systematic* W/2 (review* OR overview*)) OR (methodologic* W/2 (review* OR overview*))) OR ((quantitative W/2 (review* OR overview* OR synthes*)) OR (research W/2 (integrati* OR overview*))) OR ((integrative W/2 (review* OR overview*)) OR (collaborative W/2 (review* OR overview*)) OR (pool* W/2 analy*)) OR (data PRE/0 (synthes* OR extraction* OR abstraction*)) OR (handsearch* OR hand‐search*) OR (“mantel Haenszel” OR peto OR “der simonian” OR dersimonian OR (fixed PRE/0 effect*) OR (latin PRE/0 square*)) OR (met‐analy* OR metanaly* OR hta OR htas OR (technology PRE/0 (overview* OR appraisal* OR assessment*))) OR (meta‐regression* OR metaregression*) OR (comparative W/2 (efficacy OR effectiveness)) OR (“outcomes research” OR “relative effectiveness”) OR ((indirect OR “indirect treatment” OR mixed‐treatment) PRE/0 comparison*) OR (meta‐ethnograph* OR metaethnograph* OR “meta ethnograph” OR meta‐study OR metastudy OR “meta study”) OR ((qualitative W/2 (review* OR overview* OR synthes*)) OR (research W/2 (integrati* OR overview*))) OR (evidence W/1 map*) OR ((evidence OR realist) W/2 (review* OR overview* OR synthes*)))) OR (TITLE‐ABS‐KEY ((meta‐analy* OR metaanaly* OR (systematic PRE/0 review*) OR (biomedical PRE/0 technology PRE/0 assessment*) OR (bio‐medical PRE/0 technology PRE/0 assessment*)) OR (meta‐analys*s OR (systematic PRE/0 review*))) OR INDEXTERMS ((meta‐analy* OR metaanaly* OR (systematic PRE/0 review*) OR (biomedical PRE/0 technology PRE/0 assessment*) OR (bio‐medical PRE/0 technology PRE/0 assessment*)))) OR (SRCTYPE (meta‐analysis) OR SRCTITLE (cochrane OR (health* W/0 technology W/0 assessment*) OR (evidence PRE/0 report*))) OR (TITLE‐ABS ((medline OR cochrane OR pubmed OR medlars OR embase OR cinahl)) OR INDEXTERMS ((medline OR cochrane OR pubmed OR medlars OR embase OR cinahl))))

EMBASE

1. “patient participation”/or “stakeholder engagement”/or “participatory research”/44546
2. “community based participatory research”.ti,ab. or (“consumer”/and (participat* or engag*).ti,ab,kw.)9722
3. (coproduction or co production).ti,ab,kw.3302
4. ((stakeholder* or advisor* or reference* or expert* or consult* or steering) adj2 (group* or panel* or organi?ation* or body or lobb* or part*)).ti,ab,kw.100404
5. ((stakeholder* or consult* or patient* or consumer* or client* or public or caregiver* or “care giver*” or communit* or citizen* or user* or “service user*” or “end user*” or enduser* or clinician* or doctor* or physician* or nurs* or policymaker* or “policy maker*”) adj2 (engag* or involv* or input$ or collaborat* or cooperat* or advice or advise or feedback)).ti,ab,kw.197406
6. ((funder* or indust* or pharmac* or payer* or provider* or carer or carers or healthcare* or “health carer*” or healthprovider* or “health provider*” or frontline* or frontliner$ or “front liner$”) adj2 (engag* or involv* or input$ or collaborat* or cooperat*)).ti,ab,kw.23832
7. ((“lab technician*” or “laboratory technician*”) adj2 (engag* or involv* or input$ or collaborat* or cooperat*)).ti,ab,kw.13
8. 1 or 2 or 3 or 4 or 5 or 6 or 7353983
9. (“meta analysis” or “metaanalysis” or “metanalysis”).pt.0
10. ((systematic* adj3 (review* or overview*)) or (methodologic* adj3 (review* or overview*))).ti,ab.338290
11. ((quantitative adj3 (review* or overview* or synthes*)) or (research adj3 (integrati* or overview*))).ti,ab.16470
12. ((integrative adj3 (review* or overview*)) or (collaborative adj3 (review* or overview*)) or (pool* adj3 analy*)).ti,ab.49644
13. (“data synthes*” or “data extraction*” or “data abstraction*”).ti,ab.44001
14. (handsearch* or “hand search*”).ti,ab.12883
15. (“mantel haenszel” or peto or “der simonian” or dersimonian or “fixed effect*” or “latin square*”).ti,ab.43152
16. (“met analy*” or metanaly* or “technology assessment*” or hta or htas or “technology overview*” or “technology appraisal*”).ti,ab.17051
17. (“meta regression*” or metaregression*).ti,ab.15666
18. (“meta analy*” or metaanaly* or “systematic review*” or “biomedical technology assessment*” or “bio‐medical technology assessment*”).ti,ab,de.651032
19. (medline or cochrane or pubmed or medlars or embase or cinahl).ti,ab,de.393428
20. (cochrane or (health* adj2 “technology assessment*”) or “evidence report*”).jt.220
21. (“meta analys$s” or “systematic review*”).ti,ab,kw.309697
22. (comparative adj3 (efficacy or effectiveness)).ti,ab.23101
23. “outcomes research” or “relative effectiveness”).ti,ab.12992
24. (“indirect comparison*” or “indirect treatment comparison*” or “mixed treatment comparison*”).ti,ab.5459
25. (“meta ethnograph*” or metaethnograph* or “meta ethnograph” or metastudy or “meta study”).ti,ab.995
26. ((qualitative adj3 (review* or overview* or synthes*)) or (research adj3 (integrati* or overview*))).ti,ab.20904
27. (evidence adj2 (map$ or mapping)).ti,ab.1412
28. ((evidence or realist) adj3 (review* or overview* or synthes*)).ti,ab.87785
29. exp meta analysis/or “meta analysis (topic)”/299260
30. exp systematic review/or “systematic review (topic)”/386923
31. “biomedical technology assessment”/15880
32. 29 or 30 or 31548234
33. 9 or 10 or 11 or 12 or 13 or 14 or 15 or 16 or 17 or 18 or 19 or 20 or 21 or 22 or 23 or 24 or 25 or 26 or 27 or 28 or 32926260
34. 8 and 3332355

APA PSYCINFO

Set#: S1

Searched for: (“Stakeholder” AND (participat* OR engag*)) OR “Client Participation”

Set#: S2

Searched for: ti(Community‐based participatory research) OR ab(Community‐based participatory research) OR if(Community‐based participatory research)

Set#: S3

Searched for: ti(coproduction or co‐production) OR ab(coproduction or co‐production) OR if(coproduction or co‐production)

Set#: S4

Searched for: ti(((stakeholder* OR advisor* OR reference* OR expert* OR consult* OR steering*) N1 (group* OR panel* OR organi#ation* OR body OR lobb* OR part*))) OR ab(((stakeholder* OR advisor* OR reference* OR expert* OR consult* OR steering*) N1 (group* OR panel* OR organi#ation* OR body OR lobb* OR part*))) OR if(((stakeholder* OR advisor* OR reference* OR expert* OR consult* OR steering*) N1 (group* OR panel* OR organi#ation* OR body OR lobb* OR part*)))

Set#: S5

Searched for: ti(((stakeholder* OR consult* OR patient* OR consumer* OR client* OR public* OR caregiver* OR care‐giver* OR communit* OR citizen* OR user* OR service‐user* OR end‐user* OR clinician* OR doctor* OR physician* OR nurs* OR policymaker* OR policy‐maker* OR funder* OR indust* OR pharmac* OR payer* OR provider* OR carer* OR carers OR healthcare* OR health‐carer* OR healthprovider* OR health‐provider* OR frontline* OR (lab* N0 technician*) OR facult* OR frontliner# OR front‐liner#) N1 (engag* OR involv* OR input# OR collaborat* OR cooperat* OR advice OR advise OR feedback))) OR ab(((stakeholder* OR consult* OR patient* OR consumer* OR client* OR public* OR caregiver* OR care‐giver* OR communit* OR citizen* OR user* OR service‐user* OR end‐user* OR clinician* OR doctor* OR physician* OR nurs* OR policymaker* OR policy‐maker* OR funder* OR indust* OR pharmac* OR payer* OR provider* OR carer* OR carers OR healthcare* OR health‐carer* OR healthprovider* OR health‐provider* OR frontline* OR (lab* N0 technician*) OR facult* OR frontliner# OR front‐liner#) N1 (engag* OR involv* OR input# OR collaborat* OR cooperat* OR advice OR advise OR feedback))) OR mainsubject(((stakeholder* OR consult* OR patient* OR consumer* OR client* OR public* OR caregiver* OR care‐giver* OR communit* OR citizen* OR user* OR service‐user* OR end‐user* OR clinician* OR doctor* OR physician* OR nurs* OR policymaker* OR policy‐maker* OR funder* OR indust* OR pharmac* OR payer* OR provider* OR carer* OR carers OR healthcare* OR health‐carer* OR healthprovider* OR health‐provider* OR frontline* OR (lab* N0 technician*) OR facult* OR frontliner# OR front‐liner#) N1 (engag* OR involv* OR input# OR collaborat* OR cooperat* OR advice OR advise OR feedback)))

Set#: S6

Searched for: (Systematic Review) OR (Meta Analysis)

Set#: S7

Searched for: ti(((systematic* N2 (review* OR overview*)) OR (methodologic* N2 (review* OR overview*)))) OR ab(((systematic* N2 (review* OR overview*)) OR (methodologic* N2 (review* OR overview*))))

Set#: S8

Searched for: ti(((quantitative* N2 (review* OR overview* OR synthes*)) OR (research N2 (integrati* OR overview*)))) OR ab(((quantitative* N2 (review* OR overview* OR synthes*)) OR (research N2 (integrati* OR overview*))))

Set#: S9

Searched for: ti(((integrative N2 (review* OR overview*)) OR (collaborative N2 (review* OR overview*)) OR (pool* N2 analy*))) OR ab(((integrative N2 (review* OR overview*)) OR (collaborative N2 (review* OR overview*)) OR (pool* N2 analy*)))

Set#: S10

Searched for: ti((data W0 synthes*) OR (data W0 extraction*) OR (data W0 abstraction*)) OR ab((data W0 synthes*) OR (data W0 extraction*) OR (data W0 abstraction*))

Set#: S12

Searched for: ti(handsearch* OR hand‐search*) OR ab(handsearch* OR hand‐search*)

Set#: S13

Searched for: ti((mantel W0 haenszel) OR peto OR (der W0 simonian) OR dersimonian OR (fixed W0 effect*) OR (latin W0 square*)) OR ab((mantel W0 haenszel) OR peto OR (der W0 simonian) OR dersimonian OR (fixed W0 effect*) OR (latin W0 square*))

Set#: S14

Searched for: ti((met W0 analy*) OR metanaly* OR (technology W0 assessment*) OR HTA OR HTAs or (technology W0 overview*) OR (technology W0 appraisal*)) OR ab((met W0 analy*) OR metanaly* OR (technology W0 assessment*) OR HTA OR HTAs or (technology W0 overview*) OR (technology W0 appraisal*))

Set#: S15

Searched for: ti((meta W0 regression*) OR metaregression*) OR ab((meta W0 regression*) OR metaregression*)

Set#: S16

Searched for: ti(meta‐analy* OR metaanaly* OR (systematic W0 review*) OR “biomedical technology assessment” OR “biomedical technology assessments” OR “bio‐medical technology assessment” OR “bio‐medical technology assessments”) OR ab(meta‐analy* OR metaanaly* OR (systematic W0 review*) OR “biomedical technology assessment” OR “biomedical technology assessments” OR “bio‐medical technology assessment” OR “bio‐medical technology assessments”) OR if(meta‐analy* OR metaanaly* OR (systematic W0 review*) OR “biomedical technology assessment” OR “biomedical technology assessments” OR “bio‐medical technology assessment” OR “bio‐medical technology assessments”)

Set#: S17

Searched for: ti((medline OR cochrane OR pubmed OR medlars OR embase OR cinahl)) OR ab((medline OR cochrane OR pubmed OR medlars OR embase OR cinahl)) OR if((medline OR cochrane OR pubmed OR medlars OR embase OR cinahl))

Set#: S18

Searched for: pub((cochrane OR (health* N1 (technology W0 assessment*)) OR (evidence W0 report*)))

Set#: S19

Searched for: ti(meta‐analyst#s OR (systematic W0 review*)) OR ab(meta‐analyst#s OR (systematic W0 review*)) OR if(meta‐analyst#s OR (systematic W0 review*))

Set#: S20

Searched for: ti((comparative N2 (efficacy OR effectiveness))) OR ab((comparative N2 (efficacy OR effectiveness)))

Set#: S21

Searched for: ti(“outcomes research” OR “relative effectiveness”) OR ab(“outcomes research” OR “relative effectiveness”)

Set#: S22

Searched for: ti(((indirect OR indirect‐treatment OR mixed‐treatment) W0 comparison*)) OR ab(((indirect OR indirect‐treatment OR mixed‐treatment) W0 comparison*))

Set#: S23

Searched for: ti((meta‐ethnograph* OR metaethnograph* OR meta ethnograph OR meta‐study OR metastudy OR meta study)) OR ab((meta‐ethnograph* OR metaethnograph* OR meta ethnograph OR meta‐study OR metastudy OR meta study))

Set#: S24

Searched for: ti(((qualitative* N2 (review* OR overview* OR synthes*)) OR (research N2 (integrati* OR overview*)))) OR ab(((qualitative* N2 (review* OR overview* OR synthes*)) OR (research N2 (integrati* OR overview*))))

Set#: S25

Searched for: ti(evidence N1 map*) OR ab(evidence N1 map*)

Set#: S26

Searched for: ti(((evidence OR realist) N2 (review* OR overview* OR synthes*))) OR ab(((evidence OR realist) N2 (review* OR overview* OR synthes*)))

Set#: S27

Searched for: ((“Stakeholder” AND (participat* OR engag*)) OR “Client Participation”) OR (ti(Community‐based participatory research) OR ab(Community‐based participatory research) OR if(Community‐based participatory research)) OR (ti(coproduction OR co‐production) OR ab(coproduction OR co‐production) OR if(coproduction OR co‐production)) OR (ti(((stakeholder* OR advisor* OR reference* OR expert* OR consult* OR steering*) N1 (group* OR panel* OR organi#ation* OR body OR lobb* OR part*))) OR ab(((stakeholder* OR advisor* OR reference* OR expert* OR consult* OR steering*) N1 (group* OR panel* OR organi#ation* OR body OR lobb* OR part*))) OR if(((stakeholder* OR advisor* OR reference* OR expert* OR consult* OR steering*) N1 (group* OR panel* OR organi#ation* OR body OR lobb* OR part*)))) OR (ti(((stakeholder* OR consult* OR patient* OR consumer* OR client* OR public* OR caregiver* OR care‐giver* OR communit* OR citizen* OR user* OR service‐user* OR end‐user* OR clinician* OR doctor* OR physician* OR nurs* OR policymaker* OR policy‐maker* OR funder* OR indust* OR pharmac* OR payer* OR provider* OR carer* OR carers OR healthcare* OR health‐carer* OR healthprovider* OR health‐provider* OR frontline* OR (lab* N0 technician*) OR facult* OR frontliner# OR front‐liner#) N1 (engag* OR involv* OR input# OR collaborat* OR cooperat* OR advice OR advise OR feedback))) OR ab(((stakeholder* OR consult* OR patient* OR consumer* OR client* OR public* OR caregiver* OR care‐giver* OR communit* OR citizen* OR user* OR service‐user* OR end‐user* OR clinician* OR doctor* OR physician* OR nurs* OR policymaker* OR policy‐maker* OR funder* OR indust* OR pharmac* OR payer* OR provider* OR carer* OR carers OR healthcare* OR health‐carer* OR healthprovider* OR health‐provider* OR frontline* OR (lab* N0 technician*) OR facult* OR frontliner# OR front‐liner#) N1 (engag* OR involv* OR input# OR collaborat* OR cooperat* OR advice OR advise OR feedback))) OR mainsubject(((stakeholder* OR consult* OR patient* OR consumer* OR client* OR public* OR caregiver* OR care‐giver* OR communit* OR citizen* OR user* OR service‐user* OR end‐user* OR clinician* OR doctor* OR physician* OR nurs* OR policymaker* OR policy‐maker* OR funder* OR indust* OR pharmac* OR payer* OR provider* OR carer* OR carers OR healthcare* OR health‐carer* OR healthprovider* OR health‐provider* OR frontline* OR (lab* N0 technician*) OR facult* OR frontliner# OR front‐liner#) N1 (engag* OR involv* OR input# OR collaborat* OR cooperat* OR advice OR advise OR feedback))))

Set#: S28

Searched for: ((Systematic Review) OR (Meta Analysis)) OR (ti(((systematic* N2 (review* OR overview*)) OR (methodologic* N2 (review* OR overview*)))) OR ab(((systematic* N2 (review* OR overview*)) OR (methodologic* N2 (review* OR overview*))))) OR (ti(((quantitative* N2 (review* OR overview* OR synthes*)) OR (research N2 (integrati* OR overview*)))) OR ab(((quantitative* N2 (review* OR overview* OR synthes*)) OR (research N2 (integrati* OR overview*))))) OR (ti(((integrative N2 (review* OR overview*)) OR (collaborative N2 (review* OR overview*)) OR (pool* N2 analy*))) OR ab(((integrative N2 (review* OR overview*)) OR (collaborative N2 (review* OR overview*)) OR (pool* N2 analy*)))) OR (ti((data W0 synthes*) OR (data W0 extraction*) OR (data W0 abstraction*)) OR ab((data W0 synthes*) OR (data W0 extraction*) OR (data W0 abstraction*))) OR (ti(handsearch* OR hand‐search*) OR ab(handsearch* OR hand‐search*)) OR (ti((mantel W0 haenszel) OR peto OR (der W0 simonian) OR dersimonian OR (fixed W0 effect*) OR (latin W0 square*)) OR ab((mantel W0 haenszel) OR peto OR (der W0 simonian) OR dersimonian OR (fixed W0 effect*) OR (latin W0 square*))) OR (ti((met W0 analy*) OR metanaly* OR (technology W0 assessment*) OR HTA OR HTAs OR (technology W0 overview*) OR (technology W0 appraisal*)) OR ab((met W0 analy*) OR metanaly* OR (technology W0 assessment*) OR HTA OR HTAs OR (technology W0 overview*) OR (technology W0 appraisal*))) OR (ti((meta W0 regression*) OR metaregression*) OR ab((meta W0 regression*) OR metaregression*)) OR (ti(meta‐analy* OR metaanaly* OR (systematic W0 review*) OR “biomedical technology assessment” OR “biomedical technology assessments” OR “bio‐medical technology assessment” OR “bio‐medical technology assessments”) OR ab(meta‐analy* OR metaanaly* OR (systematic W0 review*) OR “biomedical technology assessment” OR “biomedical technology assessments” OR “bio‐medical technology assessment” OR “bio‐medical technology assessments”) OR if(meta‐analy* OR metaanaly* OR (systematic W0 review*) OR “biomedical technology assessment” OR “biomedical technology assessments” OR “bio‐medical technology assessment” OR “bio‐medical technology assessments”)) OR (ti((medline OR cochrane OR pubmed OR medlars OR embase OR cinahl)) OR ab((medline OR cochrane OR pubmed OR medlars OR embase OR cinahl)) OR if((medline OR cochrane OR pubmed OR medlars OR embase OR cinahl))) OR pub((cochrane OR (health* N1 (technology W0 assessment*)) OR (evidence W0 report*))) OR (ti(meta‐analyst#s OR (systematic W0 review*)) OR ab(meta‐analyst#s OR (systematic W0 review*)) OR if(meta‐analyst#s OR (systematic W0 review*))) OR (ti((comparative N2 (efficacy OR effectiveness))) OR ab((comparative N2 (efficacy OR effectiveness)))) OR (ti(“outcomes research” OR “relative effectiveness”) OR ab(“outcomes research” OR “relative effectiveness”)) OR (ti(((indirect OR indirect‐treatment OR mixed‐treatment) W0 comparison*)) OR ab(((indirect OR indirect‐treatment OR mixed‐treatment) W0 comparison*))) OR (ti((meta‐ethnograph* OR metaethnograph* OR meta ethnograph OR meta‐study OR metastudy OR meta study)) OR ab((meta‐ethnograph* OR metaethnograph* OR meta ethnograph OR meta‐study OR metastudy OR meta study))) OR (ti(((qualitative* N2 (review* OR overview* OR synthes*)) OR (research N2 (integrati* OR overview*)))) OR ab(((qualitative* N2 (review* OR overview* OR synthes*)) OR (research N2 (integrati* OR overview*))))) OR (ti(evidence N1 map*) OR ab(evidence N1 map*)) OR (ti(((evidence OR realist) N2 (review* OR overview* OR synthes*))) OR ab(((evidence OR realist) N2 (review* OR overview* OR synthes*))))

Set#: S29

Searched for: (((“Stakeholder” AND (participat* OR engag*)) OR “Client Participation”) OR (ti(Community‐based participatory research) OR ab(Community‐based participatory research) OR if(Community‐based participatory research)) OR (ti(coproduction OR co‐production) OR ab(coproduction OR co‐production) OR if(coproduction OR co‐production)) OR (ti(((stakeholder* OR advisor* OR reference* OR expert* OR consult* OR steering*) N1 (group* OR panel* OR organi#ation* OR body OR lobb* OR part*))) OR ab(((stakeholder* OR advisor* OR reference* OR expert* OR consult* OR steering*) N1 (group* OR panel* OR organi#ation* OR body OR lobb* OR part*))) OR if(((stakeholder* OR advisor* OR reference* OR expert* OR consult* OR steering*) N1 (group* OR panel* OR organi#ation* OR body OR lobb* OR part*)))) OR (ti(((stakeholder* OR consult* OR patient* OR consumer* OR client* OR public* OR caregiver* OR care‐giver* OR communit* OR citizen* OR user* OR service‐user* OR end‐user* OR clinician* OR doctor* OR physician* OR nurs* OR policymaker* OR policy‐maker* OR funder* OR indust* OR pharmac* OR payer* OR provider* OR carer* OR carers OR healthcare* OR health‐carer* OR healthprovider* OR health‐provider* OR frontline* OR (lab* N0 technician*) OR facult* OR frontliner# OR front‐liner#) N1 (engag* OR involv* OR input# OR collaborat* OR cooperat* OR advice OR advise OR feedback))) OR ab(((stakeholder* OR consult* OR patient* OR consumer* OR client* OR public* OR caregiver* OR care‐giver* OR communit* OR citizen* OR user* OR service‐user* OR end‐user* OR clinician* OR doctor* OR physician* OR nurs* OR policymaker* OR policy‐maker* OR funder* OR indust* OR pharmac* OR payer* OR provider* OR carer* OR carers OR healthcare* OR health‐carer* OR healthprovider* OR health‐provider* OR frontline* OR (lab* N0 technician*) OR facult* OR frontliner# OR front‐liner#) N1 (engag* OR involv* OR input# OR collaborat* OR cooperat* OR advice OR advise OR feedback))) OR mainsubject(((stakeholder* OR consult* OR patient* OR consumer* OR client* OR public* OR caregiver* OR care‐giver* OR communit* OR citizen* OR user* OR service‐user* OR end‐user* OR clinician* OR doctor* OR physician* OR nurs* OR policymaker* OR policy‐maker* OR funder* OR indust* OR pharmac* OR payer* OR provider* OR carer* OR carers OR healthcare* OR health‐carer* OR healthprovider* OR health‐provider* OR frontline* OR (lab* N0 technician*) OR facult* OR frontliner# OR front‐liner#) N1 (engag* OR involv* OR input# OR collaborat* OR cooperat* OR advice OR advise OR feedback))))) AND (((Systematic Review) OR (Meta Analysis)) OR (ti(((systematic* N2 (review* OR overview*)) OR (methodologic* N2 (review* OR overview*)))) OR ab(((systematic* N2 (review* OR overview*)) OR (methodologic* N2 (review* OR overview*))))) OR (ti(((quantitative* N2 (review* OR overview* OR synthes*)) OR (research N2 (integrati* OR overview*)))) OR ab(((quantitative* N2 (review* OR overview* OR synthes*)) OR (research N2 (integrati* OR overview*))))) OR (ti(((integrative N2 (review* OR overview*)) OR (collaborative N2 (review* OR overview*)) OR (pool* N2 analy*))) OR ab(((integrative N2 (review* OR overview*)) OR (collaborative N2 (review* OR overview*)) OR (pool* N2 analy*)))) OR (ti((data W0 synthes*) OR (data W0 extraction*) OR (data W0 abstraction*)) OR ab((data W0 synthes*) OR (data W0 extraction*) OR (data W0 abstraction*))) OR (ti(handsearch* OR hand‐search*) OR ab(handsearch* OR hand‐search*)) OR (ti((mantel W0 haenszel) OR peto OR (der W0 simonian) OR dersimonian OR (fixed W0 effect*) OR (latin W0 square*)) OR ab((mantel W0 haenszel) OR peto OR (der W0 simonian) OR dersimonian OR (fixed W0 effect*) OR (latin W0 square*))) OR (ti((met W0 analy*) OR metanaly* OR (technology W0 assessment*) OR HTA OR HTAs OR (technology W0 overview*) OR (technology W0 appraisal*)) OR ab((met W0 analy*) OR metanaly* OR (technology W0 assessment*) OR HTA OR HTAs OR (technology W0 overview*) OR (technology W0 appraisal*))) OR (ti((meta W0 regression*) OR metaregression*) OR ab((meta W0 regression*) OR metaregression*)) OR (ti(meta‐analy* OR metaanaly* OR (systematic W0 review*) OR “biomedical technology assessment” OR “biomedical technology assessments” OR “bio‐medical technology assessment” OR “bio‐medical technology assessments”) OR ab(meta‐analy* OR metaanaly* OR (systematic W0 review*) OR “biomedical technology assessment” OR “biomedical technology assessments” OR “bio‐medical technology assessment” OR “bio‐medical technology assessments”) OR if(meta‐analy* OR metaanaly* OR (systematic W0 review*) OR “biomedical technology assessment” OR “biomedical technology assessments” OR “bio‐medical technology assessment” OR “bio‐medical technology assessments”)) OR (ti((medline OR cochrane OR pubmed OR medlars OR embase OR cinahl)) OR ab((medline OR cochrane OR pubmed OR medlars OR embase OR cinahl)) OR if((medline OR cochrane OR pubmed OR medlars OR embase OR cinahl))) OR pub((cochrane OR (health* N1 (technology W0 assessment*)) OR (evidence W0 report*))) OR (ti(meta‐analyst#s OR (systematic W0 review*)) OR ab(meta‐analyst#s OR (systematic W0 review*)) OR if(meta‐analyst#s OR (systematic W0 review*))) OR (ti((comparative N2 (efficacy OR effectiveness))) OR ab((comparative N2 (efficacy OR effectiveness)))) OR (ti(“outcomes research” OR “relative effectiveness”) OR ab(“outcomes research” OR “relative effectiveness”)) OR (ti(((indirect OR indirect‐treatment OR mixed‐treatment) W0 comparison*)) OR ab(((indirect OR indirect‐treatment OR mixed‐treatment) W0 comparison*))) OR (ti((meta‐ethnograph* OR metaethnograph* OR meta ethnograph OR meta‐study OR metastudy OR meta study)) OR ab((meta‐ethnograph* OR metaethnograph* OR meta ethnograph OR meta‐study OR metastudy OR meta study))) OR (ti(((qualitative* N2 (review* OR overview* OR synthes*)) OR (research N2 (integrati* OR overview*)))) OR ab(((qualitative* N2 (review* OR overview* OR synthes*)) OR (research N2 (integrati* OR overview*))))) OR (ti(evidence N1 map*) OR ab(evidence N1 map*)) OR (ti(((evidence OR realist) N2 (review* OR overview* OR synthes*))) OR ab(((evidence OR realist) N2 (review* OR overview* OR synthes*)))

## Appendix 5 Decision support trees for study selection

**Decision tree for supporting include/exclude decisions (see next page for papers reporting Guidelines):**

**If the paper is a report of a guideline:**

## Appendix 6 Data extraction domains and codes

This appendix includes details of all domains and coding used for data extraction from evidence syntheses included within the scoping review:

- Tables 6–1: Data extraction domains and coding for all included publications
- Tables 6–2: Data extraction codes for Topic/focus of evidence synthesis domain
- Tables 6–3: Additional data extraction for “Green” studies
- Tables 6–4: Stages of a review

(*Note:* Blue text reflects changes/additions to protocol; Black text reflects items listed in protocol)

**Table 6‐1:** Data extraction domains and coding for all included publications.

Data extraction item	Extraction/coding details
Bibliographic information	Full citation
Study author	First author surname
Year of publication	Year of publication
Type of publication	Coded as: Full paper (journal publication) Abstract Dissertation or thesis Other
Type of paper	Coded as: Evidence synthesis Report of engagement in evidence synthesis Report of guideline/recommendation (where the evidence synthesis is reported as part of a guideline/recommendation) Description of methods of engagement Other
Stated aim/objective	Relevant quotation from paper, or summary of text from paper
Topic/focus of evidence synthesis	Categorized according to focus on a specific disease or health area (based on ICD‐11 headings*) or, if the focus was not on a specific disease or health area, categorized according to type of intervention (based on ICHI headings**) or as focussed on research methods, or “other.”
Methodological focus/study methodology	Relevant quotation from paper (e.g., methods section from abstract)
Type of evidence synthesized	Evidence synthesis methodology coded as: Scoping review Systematic review of intervention effectiveness (with or without meta‐analysis) Systematic review of other quantitative evidence (e.g., prevalence, patterns of recovery, diagnostic test accuracy) Realist review Meta‐ethnography Qualitative evidence synthesis (any other approach) Mixed‐method evidence synthesis Unclear Other
Why were they engaged? (Aim of engagement)	Relevant quotation from paper, relating to why interested people and groups were engaged
Description of reported method(s) or approach(es) to engagement of interested people and groups	Relevant quotations from paper, giving overview of methods of engaging interested people and groups.
Terminology used to describe methods of engagement	Noted where this was: “Expert” panel or group or team “Advisory” panel or group or team “Reference” panel or group or team “Patient,” or “Public” or “PPI” panel or group or team “Consensus” conference or meeting “Delphi” approach “Voting” or “Ranking” “Participatory” method or approach “Co‐production” or “Co‐design” (or other relevant “Co‐” term) Other
Who was engaged?	Total number and numbers of: – Patients and members of the public – Professionals – Researchers – Others
Details/experience of people engaged	Relevant quotations or extract from paper, describing who was engaged (background, numbers, etc.)
Where were they engaged? (Country)	Country of people engaged or, if this was not stated, country of lead author (from contact details)
When were people engaged?	At which, one(s) of the 12 review stages, people were engaged (See Stages of a review table, below).
How were people engaged? (approach)	Coded as: One‐time (“an approach that engages interest‐holders at a specific stage of a review, for example, when developing the question for the review, or writing lay‐summaries of a completed review. One‐time engagement can occur at just one stage or at multiple stages in the review process.”) Continuous (“the engagement of the same interest‐holders throughout the entire review process”) Combined (“the engagement of two (or more) distinct groups of interest‐holders, with some having continuous engagement…. while others had [one‐time] input at …. specific stages of the review”) Unclear/not reported
How were people engaged? (method of interaction?	Coded as (check all that apply): Direct – group (i.e., direct person‐to‐person interaction, such as meetings, Zoom calls, with more than two people) Direct – one‐to‐one (i.e., direct person‐to‐person interaction, such as meetings, Zoom calls, involving just one person and a researcher) Indirect (i.e., surveys, emails, with no direct person‐to‐person interaction) Unclear

**Table 6‐2:** Data extraction codes for the topic/focus of the evidence synthesis domain.

*International Statistical Classification of Diseases and Related Health Problems 11th Revision (ICD‐11) headings (https://icd.who.int/browse11/l-m/en):
01 Certain infectious and parasitic diseases
02 Neoplasms
03 Diseases of the blood and blood‐forming organs
04 Diseases of the immune system
05 Endocrine, nutritional, and metabolic diseases
06 Mental, behavioral or neurodevelopmental disorders
07 Sleep‐wake disorders
08 Diseases of the nervous system
09 Diseases of the visual system
10 Diseases of the ear or mastoid process
11 Diseases of the circulatory system
12 Diseases of the respiratory system
13 Diseases of the digestive system
14 Diseases of the skin
15 Diseases of the musculoskeletal system or connective tissue
16 Diseases of the genitourinary system
17 Conditions related to sexual health
18 Pregnancy, childbirth or the puerperium
19 Certain conditions originating in the perinatal period
20 Developmental anomolies
21 Symptoms, signs or clinical findings, not elsewhere classified
22 Injury, poisoning or certain other consequences of external causes
23 External causes of morbidity or mortality
24 Factors influencing health status or contact with health services
25 Codes for special purposes (covers new diseases of uncertain etiology)
26 Supplementary Chapter Traditional Medicine Conditions
**Derived from International Classification of Health Interventions (ICHI) headings (https://icd.who.int/dev11/l-ichi/en):
Interventions on health‐related behaviors
Interventions on body systems or functions
Interventions on activities and participation domains

**Table 6‐3:** Additional data extraction for “Green” studies.

How were people invited to be engaged:	Coded as: Open recruitment (“‘Open’ recruitment refers to providing opportunities for engagement through advertisement to the general population, allowing anyone to volunteer to get engaged.”) Open, fixed (“once group members had volunteered, the membership remains the same” Open, flexible (“where different people attend different events or contribute to different activities”) Closed recruitment (“‘closed’ (or ‘targeted’) recruitment strategies focus on inviting only specific people to participate”) Closed, invitation (“invitations of known individuals or recognised experts”) Closed, existing group (“recruitment from membership of an existing group”) Closed, purposive sampling (“purposive sampling to achieve representation of people with key pre‐determined characteristics, experience or expertise.”) Other Unclear/not reported
Who was engaged?	Coded as yes, no or unclear for each of the 11 types of interested people and groups: 1. patients and caregivers, 2. public, 3. providers of care, 4. policy makers, 5. program managers, 6. payers of health research, 7. payers of health services, 8. peer review editors, 9. product makers, 10. producers and commissioners, 11. principal investigators. These have been fully defined elsewhere (Petkovic et al., intro paper). Numbers of people in each category recorded.
Who was engaged? PROGRESS‐Plus characteristics	Coded as yes, no, unclear for reporting information relating to the following characteristics of people engaged: Place of residence Race/ethnicity/culture/language Occupation Gender/sex Religion Education Socioeconomic status Social capital Age Disability Other personal characteristics Free text box to record relevant information.
Who was engaged? Geographical location of people engaged.	Were people engaged from: East Asia and the Pacific Europe and Central Asia Latin America and the Caribbean Middle East and North Africa North America South Asia Sub‐Saharan Africa Coded using data categories from the World Bank.
Who was engaged? Country income level.	Were people engaged from countries that are considered: Low income Low middle income Upper middle income High income Coded using data categories from the World Bank.
Methods: form of engagement	Recorded according to the stages of the review process (see below) – Select code which best describes activity: – Meeting – Workshop – Public event – Interview(s)/focus group(s) – Survey – Other form of electronic‐based activity – Mix of more than one of above – Other form of activity (not stated above)
Methods used to facilitate of engagement	Recorded according to the stages of the review process (see below) – Select code which best describes methods: – Delphi – Nominal Group Technique – Voting or ranking – “Consultation” or group discussion – Other method – No formal methods used – No information provided
Form of governance	Coded as: – People engaged became part of the team conducting the review (sharing power with researchers) – People engaged were an advisory or steering group, contributing to the review process/project (but with limited power) – Researchers engaged with interest‐holders, but interest‐holders did not have any other contributions to the review/project (no power over wider review process) – Other – Unclear
Amount of engagement	Recording: Number of meetings (total) Length of meetings (as reported by authors) Overall length of the review period with engagement Or other relevant details relating to amount.
	This will be recorded according to the stages of the review process (see below)
At what stage in the review process did engagement occur?	Coded as yes, no, unclear for engagement at any of the 12 stages described in the stages of review table, below. Free text box to record relevant information about what happened at which stage.
What was the level of engagement (at each stage)	For each stage (as above), coded as: – Interest‐holders had overall control over final decisions/outcomes for this activity – Interest‐holders had some influence over final decisions/outcomes for this activity – Interest‐holders had no control or influence over final decisions/outcomes for this activity – The level of control/influence is not stated/is unclear – Other – Insufficient information
Ethical approval	Was ethical approval sought and reported?
Compensation	– Were expenses paid? – Were people engaged given any other compensation (financial payment, voucher, other reward)? – Were people engaged as co‐authors on the review? – Were people engaged and acknowledged within the review?
Tool or method of reporting engagement	Details of tool, framework, or checklist Was ACTIVE Framework used/referred to? (Y/N/U) Was GRIPP2 guidance used/referred to? (Y/N/U)
Other descriptions of processes to facilitate engagement	For example, could be terms of reference, contracts, training, safe spaces, timing of calls to meet availability of people and groups/geography, and so forth
Reflexivity	Do authors report using a process of reflexivity (Y/N/U) Description of any reflexivity
Conflicts of interest	Are conflicts of interest of review authors reported? (Y/N/U) Are any conflicts of interest of people engaged reported (Y/N/U)
	Description of any reporting of conflicts of interest

**Table 6‐4:** Stages of a review.

Phase of review	Key stage *(from ACTIVE framework)*	Review tasks and activities
A. Review planning and development	1. Develop question	Idea generation Priority setting specific to review development Form PICO (or alternative)
	2. Plan methods	Develop review methods
	3. Develop search *(previously no 4)*	Select databases, websites, organizations, references lists, and other sources Develop a search strategy
B. Review conduct	4. Write and publish protocol *(previously no 3)*	Draft protocol Peer review of methods and search Edit/revise protocol Publish protocol
	5. Run search	Run search De‐duplication
	6. Select studies	Apply the eligibility criteria Title/abstract screening Full text screen Consensus discussion
	7. Collect data	Extract data from included studies
	8. Assess risk of bias	Critical appraisal/risk of bias assessment of included studies
	9. Analyze data	Data synthesis (qualitative data analysis; thematic analysis) Meta‐analysis Data presentation Assess certainty (GRADE) Explore heterogeneity (sensitivity and subgroup analyses)
	10. Interpret findings	Narrative summary of findings Limitations of evidence Implications for practice, policy, future research
C. Review publication and dissemination	11. Write and publish	Draft review Peer review Edit/revise review Publish review
	12. Knowledge translation and impact	Activities to disseminate reviews – social media, conference presentation, products (infographics, videos, etc.)

## Appendix 7 Narrative summaries, according to type of evidence synthesis in 51 “Green” evidence syntheses

**Table:**

	Who was involved and how were they recruited?	What happened and when?	How much control did people engaged have over the ES?
Quantitative evidence syntheses (*n* = 10)	Seven evidence syntheses reported on the involvement of patient representatives only [64, 72, 93, 173, 179, 182, 300]. The minimum number of patient representatives involved was 2 and maximum 13 (mean: 5). Three evidence syntheses reported on the engagement of multiple interest‐holder groups: one involved 5 patients and 4 healthcare professionals [164], another involved 2 patients, 5 caregivers, 22 geriatricians, and 4 policymakers/healthcare managers [282], and one (guideline development) involved 9 providers (clinicians and clinical microbiologists) and 10 researchers who were not part of the core team [158]. Most evidence syntheses reported that they recruited interest‐holders via closed recruitment, such as through existing contacts and networks [64, 72, 93, 164, 282, 300].	Four quantitative reviews included engaged patients as co‐authors [64, 158, 173, 182]. In four evidence syntheses, interest‐holders were involved in multiple activities from question development to dissemination [64, 158, 182, 300]. Three evidence syntheses reported involvement in the early stages of the project, that is, question development and methods planning [179] and outcome prioritization [72, 282]. Three evidence syntheses employed a “top and tail” approach, where interest‐holders contributed to planning methods and the interpretation of results [93, 164, 173]. Most evidence syntheses involved discussion via meetings (mostly group‐based and online, where stated) and sometimes also Individual/written online communication [93, 158, 164, 173, 179, 182]. In addition, two studies reported 1:1 meetings [173, 182]. One study reported that they met regularly face‐to‐face and corresponded via email [64]. Three studies involved workshops [72, 179, 300]. One of these also involved ranking of outcomes at home [72], and one also involved two surveys [282].	In six quantitative reviews, we considered that the engaged people became part of the team and shared power with the review team [64, 93, 158, 164, 179, 182]. In the remaining four reviews [72, 173, 282, 300], the people engaged were considered to have an advisory role. For example, they provided feedback at certain stages of the review process, but with limited power.
Qualitative and mixed method evidence syntheses (*n* = 8)	Across all evidence syntheses, the total number of people engaged ranged from 2 to 21. All evidence syntheses involved people with lived experience as patients, carers or members of the public; in four, these were the only group of people engaged [153, 175, 229, 323], and in four, other professionals were also involved [136, 157, 222, 268].	Six of the eight evidence syntheses involved people with lived experience as co‐authors. In one of these [229], the main engagement comprised the involvement of two patient co‐authors. In four, there was another form of engagement in addition to the involvement of patient co‐authors: Gavin et al. involved people in three different ways: a patient co‐author (*n* = 1), a project steering group (*n* = 6), and a cohort of public and professional partners (*n* = 16)[136]; Hannigan et al. involved a person with lived experience as a co‐investigator/co‐author and also formed a Stakeholder Advisory Group (*n* = 13) [157]; Hallett et al. had a PPI co‐author (*n* = 1) and a PPI Reference Group (*n* = 5) [153]. In two evidence syntheses, some (but not all) members of a group are listed as co‐authors: Welch et al. had a PPI group with five members, one of which reviewed the manuscript and was a co‐author [323], and Merner et al. had a “stakeholder panel” comprising 18 people, of whom some (but not all) were listed as co‐authors [222]. Four of the eight reviews reported that they had continuous engagement throughout the review, but also had other engagement activities for specific review tasks [136, 153, 222, 268], one had two patient partners who attended weekly meetings and had active contributions at several stages of the review [229], two had a “top and tail” approach [157, 175], and one only had engaged during the final data synthesis, interpretation and publication stages [323]. None of these reviews reported the use of any formal research methods or techniques to engage people, with “meetings” being the most common form of engagement.	In some instances where the people engaged were co‐authors or co‐investigators, there is a suggestion that the people involved had control over final decisions or actions relating to the review. This is the case of Hannigan et al. [157], and possibly for Merner et al. [222]. Although Moody et al. [229] describe the co‐authors as “sharing the power” with the other review team members, it is considered more likely that the people involved were influencing, rather than controlling, decisions. In all other reviews, we judge that the people engaged had some influence, rather than overall control, although often the evidence to support these conclusions is limited.
Scoping reviews (*n* = 13)	Most scoping reviews (*n* = 7) included mixed interest‐holder groups [82, 217, 240, 302, 315, 317, 320]. Interest‐holder groups included patients, people with lived experience, caregivers/supporters, consumers, knowledge intermediaries, healthcare professionals, healthcare service providers, health service leads, clinician scientists, and researchers. Three scoping reviews did not engage any patient/public, only engaging other interest‐holders [50, 71, 167], while 3 engaged patient/public only [236, 246, 329]. The minimum number of interest‐holders engaged was 1 and maximum 20 (median 10). Most commonly, interest‐holders were recruited using closed recruitment strategies, such as through existing contacts and networks [217, 315, 317, 320, 329]. The method of recruitment was not clear for two scoping reviews [82, 240].	Six of the 13 scoping reviews included patients and/or caregivers as authors of the scoping review [217, 236, 246, 315, 317, 329]. Eight scoping reviews had an advisory committee, transdisciplinary team or patient/family partner(s) that were involved throughout the review [50, 82, 217, 236, 246, 315, 317, 329]. One of these also included a consultation with a separate group of expert researchers at the beginning of the review [317], and two included consultation with a separate group of interest‐holders at the final stage of the review [246, 329]. Three scoping reviews included consultation at the final stage of the review only [71, 302, 320]. One scoping review used a “top and tail” approach with consultation at two time points (the beginning and end of the review) with two groups of interest‐holders [240]. One scoping review included rating by interest‐holders of potential topics to address evidence synthesis gaps [167]. Five studies used meetings or a mixture of meetings and electronic communication to engage interest‐holders [217, 246, 315, 317, 329]. Four studies included interviews or focus groups [71, 240, 302, 320], and two used electronic communication only [50, 82].	The people engaged became part of the team and shared power sufficient for authorship on the scoping review with the researchers in six scoping reviews [217, 236, 246, 315, 317, 329], with two instances where patients also had leadership roles on the review team [317, 329]. In two others, interest‐holders contributed to the project but with limited control or contribution to the review process [50, 302]; in four interest‐holders only provided input with no control or contribution to the review process [71, 167, 240, 320]; and it was unclear in one scoping review [82].
Realist reviews (*n* = 14)	Thirteen out of 14 realist reviews engaged both topic experts and patients or carers [74, 98, 117, 127, 146, 178, 224, 242, 257, 266, 270, 331, 332] whilst one review [181] only included providers or other professional topic experts. In eight realist reviews, the method of recruitment was unclear. The other reviews (*n* = 6) reported closed recruitment often using purposive sampling of existing contacts and networks [74, 146, 178, 181, 224, 266].	Nine realist reviews included engaged people as co‐authors. Unlike other types of evidence synthesis, realist reviews do not follow a linear process from literature searching to data extraction and analysis. All the included realist reviews reported engagement with topic experts and/or patient and public representatives to generate initial program theories. For example, John et al. [178], Millar et al. [224], and Bunn et al. [74] conducted individual interviews with relevant interest‐holders, whilst other reviews reported using workshops and meetings to gather data and develop the initial theories that would guide the rest of the realist review process [266, 332]. The other main activity in which people were engaged was to refine and validate the final program theory. Engagement in this activity was reported in all the realist reviews. Interest‐holders were often asked to provide feedback on the preliminary analysis (or Context‐mechanism‐outcome configurations [CMOCs] in realist reviews) and to test or refine the program theories that were developed from the analysis. This was done primarily through interviews, workshops, and team meetings.	In seven realist reviews, the people engaged became part of the team [98, 181, 224, 242, 266, 270, 332]. In all these reviews, plus two further reviews [117, 257], some or all the engaged people were included as co‐authors. In six reviews, the engaged people had an advisory role [74, 117, 127, 146, 178, 257]. In Millar et al. [224], the control held by the engaged people of the review process was unclear.
Overviews of reviews (*n* = 3)	Two of these held meetings with the people engaged using a “top and tail” approach (meetings before and after conducting the review). One engaged a mixed group of patients/caregivers and providers [296], while the other held three meetings with a patient group, but also engaged other interest‐holders throughout the review via informal conversations, emails, and virtual meetings [102]. The third overview [63] primarily engaged people at the planning stage, through conducting key informant interviews. Some of the key informants also provided a peer review of the final review. The total number of people engaged in these 3 overviews ranged from 11 to 14. The majority of those engaged appeared to be providers, with a smaller number of patients reported to be engaged, although the exact number of people from each interest‐holder group was not always clear.		
Projects involving multiple reviews (*n* = 3)	Reeve et al. [262] report on a scoping review and realist synthesis. Planned engagement across the project included 4 PPI events/workshops, a Stakeholder Group (meeting twice/year), plus a Project Working Group (delivering day‐to‐day tasks). Difficulties with recruitment and engagement meant that, instead, there was one PPI co‐author, an Academic Advisory Group (meeting five times in total), and three interest‐holder meetings. Lourida et al. [206] conducted three systematic reviews focussed on experiences of care by people with dementia. They formed a project advisory group (PAG) that was engaged through four face‐to‐face meetings and by email. Meetings were attended by between 12 and 17 people, including patients/caregivers, principal investigators, commissioners, and providers. Some of the PAG were co‐authors, and others were not. Details of all meetings and activities were provided. One of the meetings used Lego as a creative tool to facilitate engagement. Walker et al. [313] was a report of patient engagement in a series of systematic reviews focussed on interventions to improve the mental health of children and young people with long‐term physical conditions. A closed group of eight children and young people, at times joined by their parents, had four face‐to‐face meetings with the research team during the project.		

## Appendix 8 Summary of when the evidence synthesis engagement was reported to occur within the 51 “green” evidence syntheses

**Table:**

Stage of review	Review activities	Total number of reported engagement activities (from 51 ES)	Quantitative ES	Realist reviews	Scoping reviews	Papers reporting multiple reviews	Overviews of reviews	Qualitative or mixed method ES
1. Develop question	Idea generation	11	3	1	3	0	1	3
	Priority setting^a^	4	2	0	1	0	0	1
	Form PICO (or alternative)	26	7	11	3	1	0	4
2. Plan methods	Plan general methods	21	7	3	0	4	2	5
	Plan inclusion criteria	16	7	2	0	1	3	3
	Data extraction plan	13	3	2	3	0	3	2
	Data analysis plan	6	4	0	0	0	1	1
3. Develop search	Select databases, etc.^b^	7	0	3	1	0	0	3
	Develop search strategy	15	3	2	4	1	0	5
4. Write and publish protocol	Draft protocol	7	2	1	0	0	0	4
	Peer review of methods and search	5	0	0	2	1	0	2
	Edit/revise protocol	7	2	0	2	0	0	3
	Publish protocol	4	2	0	0	0	0	2
5. Run search	Run search (databases)	1	0	0	0	0	0	1
	Run search (gray+)	5	1	2	1	0	0	1
	De‐duplication	1	0	0	0	0	0	1
6. Select studies	Apply eligibility criteria/screening/selection	12	3	2	3	1	0	3
7. Collect data	Extract data from included studies	12	2	3	2	2	1	2
8. Assess risk of bias	Critical appraisal/risk of bias of included studies	3	1	1	0	0	0	1
9. Analyze data/Synthesis	Data synthesis^c^	31	3	12	5	1	3	7
	Assess certainty/Explore heterogeneity	6	1	0	0	1	1	3
10. Interpret findings	Interpretation and summarize findings^d^	39	7	8	11	4	0	9
11. Write and publish review	Draft review	12	4	3	2	0	0	3
	Peer review	9	0	0	4	2	1	2
	Edit/revise review	19	4	4	5	0	1	5
	Publish review	20	4	6	5	1	0	4
12. Knowledge translation and impact	Activities to disseminate reviews^e^	19	5	1	1	3	2	7

*Note:* The darker the green shade, the greater the frequency of reported engagement activities at that stage of an ES.

^a^Specific to review development; ^b^Including websites, organizations, references lists and other sources; ^c^Including qualitative data analysis; thematic analysis; quantitative meta‐analysis; data presentation; ^d^Including producing Narrative summary of findings/Limitations of evidence/Implications for practice, policy, future research recommendations; ^e^Including journal publications and reports; social media, conference presentation, products (infographics, videos, etc.).
