# Supplementary material for: Methods of Engaging Interest‐Holders in Healthcare Evidence Syntheses: A Scoping Review
Source: Cochrane Evid Synth Methods. 2026 Jan 15;4(1):e70066. doi: 10.1002/cesm.70066 (PMC12822417; doi:10.1002/cesm.70066)
Supplement: Supplementary file 3 — Supplementary Material 3, Comparison with previous scoping review results. [file CESM-4-e70066-s004.docx]

**Supplementary Material 3**

**Comparison of results of updated scoping review and previous scoping review**

| **Type of publication** | **Sub-category** | **Previous review** (unique publications, n=291) | | **Updated review** (unique publications, n=302) | | **Total unique publications (n=593)** | | **Total publications** (includes 14 publications counted twice^a,b^) |
| --- | --- | --- | --- | --- | --- | --- | --- | --- |
| Publications reporting interest-holder engagement in an evidence synthesis | Evidence Syntheses | 91 | 31.3% | 213 ^a^ | 70.3% | 304 ^a^ | 51.2% | 317 |
|  | Reports of guideline or recommendation | 158 | 54.3% | 34 | 11.2% | 192 | 32.3% | 192 |
|  | Descriptions of methods of involving interest-holders in an evidence synthesis | 16 | 5.5% | 11 ^b^ | 4.0% | 27 ^b^ | 4.7% | 28 |
|  | *Sub-total* | *265* | *91.1%* | *258* | *85.4%* | *523* | *88.2%* | *537* |
| Publications providing an account or description of engagement, or methods of engagement, relevant to evidence syntheses | | 26 | 8.9% | 44 ^a, b^ | 14.6% | 70 | 11.8% | 70 |
|  | Total | 291 | 100% | 302 | 100% | 593 | 100% | 607 |

**Table 3.1: Types of included paper**

^a^ 13 of the ‘Methods’ papers were also considered to be Evidence Syntheses; but are presented as ‘Methods’ papers within the narrative of our scoping review.

^b^ 1 of the ‘Methods’ papers was also considered to be a paper specifically describing engagement of interest-holders in ES ; but is presented as ‘Methods’ papers within the narrative of our scoping review.

.

| **Interest-holders engaged** | Previous review (n=265) | | Updated review (n=272) | | Total (n=537) | |
| --- | --- | --- | --- | --- | --- | --- |
| **Patients / carers / family / public members (with or without other interest-holders)** | 77 | 29.1% | 200 | 73.5% | 277 | 51.5% |
| **Other interest-holders (professionals) only** | 108 | 40.8% | 46 | 16.9% | 154 | 28.7% |
| **Unclear** | 80 | 30.2% | 26 | 9.6% | 106 | 19.7% |
|  | 265 | 100.0% | 272 | 100.0% | 537 | 100.0% |

**Table 3.2: Interest-holders engaged**

| **Country** | **Previous review (n=265)** | | **Updated review (n=272)** | | **Total (n=537)** | |
| --- | --- | --- | --- | --- | --- | --- |
| **UK** | 71 | 26.79% | 109 | 40.07 | 180 | 33.52 |
| **USA** | 87 | 32.83% | 53 | 19.49 | 140 | 26.07 |
| **Canada** | 25 | 9.43% | 36 | 13.24 | 61 | 11.36 |
| **Australia** | 18 | 6.79% | 17 | 6.25% | 35 | 6.52% |
| **Ireland** | 0 | 0.00% | 15 | 5.51% | 15 | 2.79% |
| **Netherlands** | 11 | 4.15% | 3 | 1.10% | 14 | 2.61% |
| **Germany** | 10 | 3.77% | 3 | 1.10% | 13 | 2.42% |
| **Italy** | 6 | 2.26% | 4 | 1.47% | 10 | 1.86% |
| **France** | 6 | 2.26% | 0 | 0.00% | 6 | 1.12% |
| **Spain** | 4 | 1.51% | 2 | 0.74% | 6 | 1.12% |
| **Japan** | 3 | 1.13% | 1 | 0.37% | 4 | 0.74% |
| **Switzerland** | 3 | 1.13% | 1 | 0.37% | 4 | 0.74% |
| **Austria** | 3 | 1.13% | 0 | 0.00% | 3 | 0.56% |
| **China** | 2 | 0.75% | 1 | 0.37% | 3 | 0.56% |
| **Denmark** | 2 | 0.75% | 1 | 0.37% | 3 | 0.56% |
| **Saudi Arabia** | 3 | 1.13% | 0 | 0.00% | 3 | 0.56% |
| **Belgium** | 2 | 0.75% | 0 | 0.00% | 2 | 0.37% |
| **Brazil** | 1 | 0.38% | 1 | 0.37% | 2 | 0.37% |
| **New Zealand** | 1 | 0.38% | 1 | 0.37% | 2 | 0.37% |
| **Norway** | 2 | 0.75% | 0 | 0.00% | 2 | 0.37% |
| **Portugal** | 0 | 0.00% | 2 | 0.74% | 2 | 0.37% |
| **Sweden** | 0 | 0.00% | 2 | 0.74% | 2 | 0.37% |
| **Multiple countries** | 0 | 0.00% | 17 | 6.25% | 17 | 3.17% |
| **One paper from each of: (previous review) Chile, Columbia, Iran, Korea, Taiwan; (updated review) Argentina, Malawi, Singapore.** | 5 | 1.89% | 3 | 1.10% | 8 | 1.49% |
| **Total** | 265 | 100.00% | 272 | 100.00% | 537 | 100.00% |

**Table 3.3: Countries from which interest-holders engaged**

| **Stage of evidence synthesis process** | **Previous review (n=265)** | | **Updated review (n=272)** | | **Total (n=537)** | |
| --- | --- | --- | --- | --- | --- | --- |
| **1. setting scope / review questions** | 7 | 2.6% | 12 | 4.4% | 19 | 3.5% |
| **2. interpreting results after review completion** | 53 | 20.0 | 39 | 14.3% | 92 | 17.1% |
| **1 and 2 (‘Top and tail’ approach’)** | 18 | 6.8% | 113 | 41.5% | 131 | 24.4% |
| **3. throughout/within the review process** | 61 | 23.0 | 36 | 13.2% | 97 | 18.1% |
| **unclear** | 126 | 47.5 | 67 | 24.6% | 193 | 35.9% |
| **Other (searching)** | 0 | 0.0% | 5 | 1.8% | 5 | 0.9% |
|  | 265 | 100% | 272 | 100% | 537 | 100% |

**Table 3.4: Stage of the evidence synthesis process at which interest-holders were engaged**

| **Focus of evidence synthesis** | **Previous review** | | **Updated review** | | **Total (n=537)** | |
| --- | --- | --- | --- | --- | --- | --- |
| **01 Certain infectious and parasitic diseases** | 16 | 6.0% | 4 | 1.5% | 20 | 3.7% |
| **02 Neoplasms** | 19 | 7.2% | 38 | 14.0% | 57 | 10.6% |
| **03 Diseases of the blood and blood-forming organs** | 4 | 1.5% | 3 | 1.1% | 7 | 1.3% |
| **04 Diseases of the immune system** | - | - | 1 | 0.4% | 1 | 0.2% |
| **05 Endocrine, nutritional and metabolic diseases** | 12 | 4.5% | 6 | 2.2% | 18 | 3.4% |
| **06 Mental, behavioural or neurodevelopmental disorders** | 25 | 9.4% | 60 | 22.1 | 85 | 15.8% |
| **07 Sleep-wake disorders** | - | - | 0 | 0.0% | 0 | 0.0% |
| **08 Diseases of the nervous system** | 5 | 1.9% | 15 | 5.5% | 20 | 3.7% |
| **09 Diseases of the visual system** | 1 | 0.4% | 2 | 0.7% | 3 | 0.6% |
| **10 Diseases of the ear or mastoid process** | 2 | 0.8% | 0 | 0.0% | 2 | 0.4% |
| **11 Diseases of the circulatory system** | 9 | 3.4% | 1 | 0.4% | 10 | 1.9% |
| **12 Diseases of the respiratory system** | 13 | 4.9% | 7 | 2.6% | 20 | 3.7% |
| **13 Diseases of the digestive system** | 13 | 4.9% | 3 | 1.1% | 16 | 3.0% |
| **14 Diseases of the skin** | 7 | 2.6% | 0 | 0.0% | 7 | 1.3% |
| **15 Diseases of the musculoskeletal system or connective tissue** | 18 | 6.8% | 10 | 3.7% | 28 | 5.2% |
| **16 Diseases of the genitourinary system** | 8 | 3.0% | 2 | 0.7% | 10 | 1.9% |
| **17 Conditions related to sexual health** | - | - | 1 | 0.4% | 1 | 0.2% |
| **18 Pregnancy, childbirth or the puerperium** | 7 | 2.6% | 4 | 1.5% | 11 | 2.0% |
| **19 Certain conditions originating in the perinatal period** | 0 | 0.0% | 1 | 0.4% | 1 | 0.2% |
| **20 Developmental anomalies** | 2 | 0.8% | 0 | 0.0% | 2 | 0.4% |
| **21 Symptoms, signs or clinical findings, not elsewhere classified** | 0 | 0.0% | 0 | 0.0% | 0 | 0.0% |
| **22 Injury, poisoning or certain other consequences of external causes** | 6 | 2.3% | 0 | 0.0% | 6 | 1.1% |
| **23 External causes of morbidity or mortality** | 2 | 0.8% | 1 | 0.4% | 3 | 0.6% |
| **24 Factors influencing health status or contact with health services** | 23 | 8.7% | 10 | 3.7% | 33 | 6.1% |
| **Interventions** | 33 | 12.5% | 45 | 16.5% | 78 | 14.5% |
| **Other** | 17 | 6.4% | 26 | 9.6% | 43 | 8.0% |
| **Research methods** | 23 | 8.7% | 32 | 11.8% | 55 | 10.2% |
| **Total** | 265 | 100% | 272 | 100% | 537 | 100% |

**Table 3.5: Focus of the included papers.**

***01-24 are ICD-11 categories (note that in the previous review ICD-10 categories were used).***

| **Previous review** | **Green** |  | **Amber** |  | **Red** |  | **Total** |
| --- | --- | --- | --- | --- | --- | --- | --- |
| Evidence synthesis | 13 | 14.3% | 32 | 35.2% | 46 | 50.5% | 91 |
| Description of methods of engagement | 15 | 93.8% | 1 | 6.3% | 0 | 0.0% | 16 |
| Report of a guideline/ recommendation | 2 | 1.3% | 44 | 27.8% | 112 | 70.9% | 158 |
| Total | 30 | 11.3% | 77 | 29.1% | 158 | 59.6% | 265 |
| **Updated review** | **Green** |  | **Amber** |  | **Red** |  | **Total** |
| Evidence synthesis | 41 | 18.1% | 114 | 50.4% | 71 | 31.4% | 226 |
| Description of methods of engagement | 9 | 75.0% | 2 | 16.7% | 1 | 8.3% | 12 |
| Report of a guideline/ recommendation | 1 | 2.9% | 26 | 76.5% | 7 | 20.6% | 34 |
| Total | 51 | 18.8% | 142 | 52.2% | 79 | 29.0% | 272 |
| **Total papers** | **Green** |  | **Amber** |  | **Red** |  | **Total** |
| Evidence synthesis | 54 | 17.0% | 146 | 46.1% | 117 | 36.9% | 317 |
| Description of methods of engagement | 24 | 85.7% | 3 | 10.7% | 1 | 3.6% | 28 |
| Report of a guideline/ recommendation | 3 | 1.6% | 70 | 36.5% | 119 | 62.0% | 192 |
| Total | 81 | 15.1% | 219 | 40.8% | 237 | 44.1% | 537 |

**Table 3.6: Comprehensiveness of description of method or approach to involvement**

| **Interest-holders engaged** | **Previous review (n=30)** | | **Updated review (n=51)** | | **Total (n=81)** | |
| --- | --- | --- | --- | --- | --- | --- |
| Patients/carers/family/  public only | 12 | 40.0% | 13 | 25.5% | 25 | 30.9% |
| Patients/carers/family/  public + other interest-holders (professionals) | 13 | 43.3% | 30 | 58.8% | 43 | 53.1% |
| Professional interest-holders only | 5 | 16.7% | 6 | 11.8% | 11 | 13.6% |
| Patients/carers/family/public but unclear if other interest-holders (professionals) | - | - | 2 | 3.9% | 2 | 2.5% |
| Total | 30 | 100% | 51 | 100% | 81 | 100% |

**Table 3.7: Summary of interest-holders engaged in ‘green’ publications**

|  |  | **Yes** | | **No – states not paid** | | **Not reported** | |
| --- | --- | --- | --- | --- | --- | --- | --- |
| **Were expenses paid? (i.e. out-of-pocket expenses such as travel, childcare or internet access)** | Previous review | 7 | 23.3% | 0 | 0.0% | 23 | 76.7% |
|  | Updated review | 9 | 17.6% | 2 | 3.9% | 40 | 78.4% |
|  | **Total** | 16 | 19.8% | 2 | 2.5% | 63 | 77.8% |
| **Were people engaged given any other compensation (financial payment, voucher, other reward)** | Previous review | 7 | 23.3% | 1 | 3.3% | 22 | 73.3% |
|  | Updated review | 11 | 21.5% | 1 | 2.0% | 39 | 76.5% |
|  | **Total** | 18 | 22.2% | 2 | 2.5% | 61 | 75.3% |

**Table 3.8: Summary of information relating to compensation provided to interest-holders reported in ‘green’ papers.**

|  | previous review | updated review | total |  | previous review | updated review | total |
| --- | --- | --- | --- | --- | --- | --- | --- |
| Key stages of an ES | n=30 | n=51 | n=81 |  | Percentage of studies with involvement | | |
| Before the review | 1 | 11 | 12 |  | 3.3% | 21.6% | 14.8% |
| 1. Develop question | 11 | 26 | 37 |  | 36.7% | 51.0% | 45.7% |
| 2. Plan methods | 7 | 21 | 28 |  | 23.3% | 41.2% | 34.6% |
| 3. Protocol | 2 | 7 | 9 |  | 6.7% | 13.7% | 11.1% |
| 4. Develop search | 4 | 15 | 19 |  | 13.3% | 29.4% | 23.5% |
| 5. Run search | 2 | 5 | 7 |  | 6.7% | 9.8% | 8.6% |
| 6. Select studies | 2 | 12 | 14 |  | 6.7% | 23.5% | 17.3% |
| 7. Collect data | 5 | 12 | 17 |  | 16.7% | 23.5% | 21.0% |
| 8. Assess ROB | 1 | 3 | 4 |  | 3.3% | 5.9% | 4.9% |
| 9. Analyse / synthesise data | 5 | 31 | 36 |  | 16.7% | 60.8% | 44.4% |
| 10. Interpret findings | 16 | 39 | 55 |  | 53.3% | 76.5% | 67.9% |
| 11. Write & publish review | 2 | 20 | 22 |  | 6.7% | 39.2% | 27.2% |
| 12. Knowledge translation | 9 | 19 | 28 |  | 30.0% | 37.3% | 34.6% |
|  | | | | | | | |
| Throughout the ES | 11 | 11 | 26 |  | 36.7% | 21.6% | 32.1% |

**Table 11: Summary of when within the ES engagement was reported to occur within the 81 ‘green’ papers. The darker the green shade the greater the proportion of ES reporting engagement at that stage of an ES.**
